# Genomic structural equation modeling reveals latent phenotypes in the human cortex with distinct genetic architecture

**DOI:** 10.1038/s41398-024-03152-y

**Published:** 2024-10-24

**Authors:** Rajendra A. Morey, Yuanchao Zheng, Henry Bayly, Delin Sun, Melanie E. Garrett, Marianna Gasperi, Adam X. Maihofer, C. Lexi Baird, Katrina L. Grasby, Ashley A. Huggins, Courtney C. Haswell, Paul M. Thompson, Sarah Medland, Daniel E. Gustavson, Matthew S. Panizzon, William S. Kremen, Caroline M. Nievergelt, Allison E. Ashley-Koch, Mark W. Logue

**Affiliations:** 1 Brain Imaging and Analysis Center, Duke University, Durham, NC 27710 USA; 2 Department of Psychiatry and Behavioral Sciences, Duke University School of Medicine, Durham, NC USA; 3 VISN 6 MIRECC, VA Health Care System, Croasdaile Drive, Durham, NC 27705 USA; 4 National Center for PTSD, VA Boston Healthcare System, Boston, MA 02130 USA; 5 Department of Biostatistics, Boston University School of Public Health, Boston, MA 02118 USA; 6 Department of Medicine, Duke Molecular Physiology Institute, Carmichael Building, Duke University Medical Center, Durham, NC 27701 USA; 7 VA Center of Excellence for Stress and Mental Health, VA San Diego Healthcare System, San Diego, CA 92161 USA; 8 Research Service VA, San Diego Healthcare System, San Diego, CA 92161 USA; 9 Department of Psychiatry, University of California San Diego, La Jolla, CA 92093 USA; 10 Psychiatric Genetics, QIMR, Berghofer Medical Research Institute, 4006 Brisbane, QLD Australia; 11 Imaging Genetics Center, Stevens Neuroimaging & Informatics Institute Keck School of Medicine University of Southern California, Los Angeles, CA 90033 USA; 12 Queensland Institute for Medical Research, Berghofer Medical Research Institute, 4006 Brisbane, QLD Australia; 13 Institute for Behavioral Genetics, University of Colorado Boulder, Boulder, CO 80303 USA; 14 Stein Institute for Research on Aging, University of California San Diego, La Jolla, CA 92093 USA; 15 Department of Psychiatry, Boston University School of Medicine, Boston, MA 02118 USA; 16 Biomedical Genetics, Boston University School of Medicine, Boston, MA 02118-2526 USA

**Keywords:** Comparative genomics, Genomics

## Abstract

Genetic contributions to human cortical structure manifest pervasive pleiotropy. This pleiotropy may be harnessed to identify unique genetically-informed parcellations of the cortex that are neurobiologically distinct from functional, cytoarchitectural, or other cortical parcellation schemes. We investigated genetic pleiotropy by applying genomic structural equation modeling (SEM) to map the genetic architecture of cortical surface area (SA) and cortical thickness (CT) for 34 brain regions recently reported in the ENIGMA cortical GWAS. Genomic SEM uses the empirical genetic covariance estimated from GWAS summary statistics with LD score regression (LDSC) to discover factors underlying genetic covariance, which we are denoting *genetically informed brain networks* (GIBNs). Genomic SEM can fit a multivariate GWAS from summary statistics for each of the GIBNs, which can subsequently be used for LD score regression (LDSC). We found the best-fitting model of cortical SA identified 6 GIBNs and CT identified 4 GIBNs, although sensitivity analyses indicated that other structures were plausible. The multivariate GWASs of the GIBNs identified 74 genome-wide significant (GWS) loci (*p* < 5 × 10^−^^8^), including many previously implicated in neuroimaging phenotypes, behavioral traits, and psychiatric conditions. LDSC of GIBN GWASs found that SA-derived GIBNs had a positive genetic correlation with bipolar disorder (BPD), and cannabis use disorder, indicating genetic predisposition to a larger SA in the specific GIBN is associated with greater genetic risk of these disorders. A negative genetic correlation was observed between attention deficit hyperactivity disorder (ADHD) and major depressive disorder (MDD). CT GIBNs displayed a negative genetic correlation with alcohol dependence. Even though we observed model instability in our application of genomic SEM to high-dimensional data, jointly modeling the genetic architecture of complex traits and investigating multivariate genetic links across neuroimaging phenotypes offers new insights into the genetics of cortical structure and relationships to psychopathology.

## Introduction

A number of different neurobiological markers have been employed in conjunction with various organizational schemes to map the human cortex. It is possible that individual differences in regional cortical surface area (SA) and cortical thickness (CT) may drive factors that affect each person and each region independently. However, the covariance structure of regional SA and CT reveals that individual differences are systematically coordinated within communities of brain regions, fluctuate in magnitude together within a population, may be instantiated as structural covariance networks (SCN) [1], and partially recapitulate established organizational schemes [2–5]. For instance, SCN organization is consistent with topological patterns of cortical maturation observed throughout developmental stages from childhood and adolescence into early adulthood [6], and the same patterns are then targeted by neurodegenerative diseases in late life [7, 8]. Second, brain regions with highly correlated CT or SA often represent networks that perform dedicated cognitive processes [1, 9, 10]. Third, regions within SCNs tend to be directly connected by white matter tracts. Indeed, about 40% of SCN connections show convergent white matter fiber connections, although other relationships captured by SCNs are distinct from fiber connectivity [5].

The correlation structure between regions represented by a SCN is influenced by both the environment and genetics. The genetic factors underlying structural correlations closely resemble functional and developmental patterns [4, 5, 11]. We will refer to these patterns of genetic correlations between brain regions as *genetically informed brain networks* (GIBNs). Genetic correlations of CT and SA regions have been examined in twin studies [12, 13]. These genetic influences were recapitulated in over 400 twin pairs, to show that the cortex is organized genetically into communities of structural and functional regions, is hierarchical, modular, and bilaterally symmetric [11]. Their genetically informed parcellation identified 12 spatially contiguous regions that qualify as GIBNs.

While twin studies have laid important groundwork regarding genetic correlations of the brain, they have several limitations. First, twin studies do not provide specific genetic variants associated with genetically correlated regions [11]. Second, twin studies rely on the *equal environment* assumption, which may be invalid for some traits. Third, quantifying the genetic correlation between CT/SA and assembling a well-powered cohort of low prevalence traits such as schizophrenia (0.5% prevalence) [14] or bipolar disorder (1% prevalence) [15] is extremely difficult due to the rarity of pairs with twins affected by one or both traits. Recently, genetic correlations between brain regions derived from GWAS results have been applied to estimate the contribution of common genetic variation to CT/SA heritability [16]. This method confers several advantages over twin studies as they do not have the same assumptions, allow effect-size estimation for individual variants, and have the ability to test genetic correlations with other traits in different populations. These SA and CT GWAS results reveal pleiotropy and genetic correlation across many neuroimaging phenotypes [17, 18].

Genomic structural equation modeling (gSEM) is a multivariate statistical method that can leverage the genetic architecture of multiple genetically correlated phenotypes to derive relatively few latent phenotypes, which describe the observed genetic correlation and elucidate loadings of multiple phenotypes onto the latent phenotype [19]. Therefore, gSEM applied to GWAS offers a genetically informed clustering of the cortex that may be neurobiologically distinct from functional and cytoarchitectural parcellations [6, 20]. Multiple regions that have significant loadings on a particular factor define the brain regions that can be described as GIBN. Importantly, gSEM can be used to estimate the strength of association between genetic variants and each latent factor in a multivariate GWAS of each GIBN using GWAS summary statistics for the individual traits. Thus, gSEM provides a description of the underlying genetic architecture of the traits being examined and effect size estimates for specific SNPs and their association with the latent factors.

In the present study, we sought to elucidate the genetic architecture of 34 regional SA and CT phenotypes reported in the ENIGMA-3 GWAS of over 50,000 primarily healthy individuals. We hypothesized that gSEM might identify cortical SA networks consistent with the clusters described by Chen et al. [11], along with other viable solutions. The genetic correlations reported in Grasby et al. [18], were stronger within major anatomical lobes than across lobes. Thus, while we predicted gross lobar structure may be reflected by GIBNs, we further predicted that GIBNs may represent regions corresponding to functional networks, canonical resting-state networks (RSN), fiber tract networks, and other neurobiological systems [6, 11]. We hypothesized that most genetic variants discovered by the ENIGMA-3 cortical GWAS would influence GIBNs. We also sought to discover novel genetic markers and links between known genetic variants and GIBNs.

Our motivation for the present analysis was that there is robust evidence of disrupted cortical structure and function for most psychiatric disorders [21, 22]. We also know psychiatric disorders are polygenic and there is significant genetic correlation between disorders [23–27]. We further hypothesized genetic correlations between GIBNs and major neuropsychiatric disorders that are stronger than correlations between global measures of SA or CT.

## Methods

### Data

We used the results of the ENIGMA-3 cortical GWAS as reported in Grasby et al. [18]. that identified genetic loci associated with variation in cortical SA and CT measures in 51,665 individuals, primarily (~94%) of European descent, from 60 international cohorts. All subjects provided informed consent to participate in study procedures approved by the local ethics board or IRB. The present study was deemed exempt by the Duke University IRB reviewed as Protocol ID: Pro00079963 and Protocol Title: Trauma and Genomics Modulate Brain Structure across Common Psychiatric Disorders. In ENIGMA-3, phenotype measures were extracted from structural MRI scans for 34 regions defined by the Desikan-Killiany atlas using gyral anatomy, which establishes coarse partitions of the cortex [28]. This study analyzed global measures of total cortical SA and average CT, as well as 34 regional measures of SA and CT averaged across left and right hemisphere structures to yield 70 distinct phenotypes. Multiple testing correction in the ENIGMA-3 GWAS was based on 70 independent phenotypes with a GWS threshold of P ≤ 8.3 × 10^−10^. We accessed the GWAS summary results for the 34-regional bilateral analyses. The primary GWAS analyses presented in Grasby et al. adjusted for global SA and mean CT. However, we utilized alternate results without global adjustments, as the global-adjusted results produce multiple negative genetic correlations between regions (see Supplementary Figs. S1 and S2), which might be artifactual and lead to uninterpretable factor loadings. Regional SA and CT metrics were analyzed separately due to computational limitations and because negative genetic correlations between SA and CT could complicate model interpretation [16, 18, 29].

### Ethics approval and consent to participate

All subjects provided informed consent to participate in study procedures approved by the local ethics board or IRB. The present study was deemed exempt by the Duke University IRB reviewed as Protocol ID: Pro00079963 and Protocol Title: Trauma and Genomics Modulate Brain Structure across Common Psychiatric Disorders.

### Analysis

Our analyses were performed using the GenomicSEM R package [19]. The gSEM was performed twice, once for 34 SA regions and once for 34 CT regions. The gSEM fitting process includes an exploratory factor analysis (EFA) stage and a confirmatory factor analysis (CFA) stage. In gSEM, these steps are usually not performed on the basis of independent cohorts. Many gSEM studies include the same GWAS data in both EFA and CFA [30–33]. To avoid overfitting, other studies split the chromosomes into two distinct sets, usually odd-numbered autosomal chromosomes and even autosomal chromosomes, and use one for EFA and the other set for CFA [34–37] and sensitivity analysis [38]. In this study, we chose the second strategy and analyzed odd chromosomes in the EFA and even chromosomes in the CFA. Whereas SEM often fits multiple models corresponding to a priori hypotheses built on theoretical models, we took a hypothesis-free (data driven) approach. In the EFA, we fit models allowing for 1 to 10 factors, for each of SA and CT. Scree plots were examined to ensure that 10 factors would be sufficient (see Figs. S3 and S4). In the EFA step, positive factor loading estimates greater than a pre-specified threshold from the EFA were carried forward to the CFA to be re-estimated, and the remaining loading parameters were set to zero [39]. As there was no consensus on factor loading cutoffs [19, 40], we tested two thresholds: 0.3 and 0.5. Cross-loadings were allowed if they exceeded the threshold. Factors that loaded on only a single region were removed as single regions do not constitute factors. Therefore, some models with a large number of factors ended up as redundant and were not carried forward to CFA as the investigation of single regions was already carried out by Grasby et al. [18]. We additionally ran a sensitivity analysis in which EFA was run using the even chromosomes and CFA was performed using the odd chromosomes to determine if order affected the final model.

The Akaike Information Criteria (AIC) was used as our primary measure of model fit. For our purposes, a model which minimized the AIC was deemed optimal. Standardized root-mean square residual (SRMR), model *χ*^2^, and Comparative Fit Index (CFI) were also calculated. A lower SRMR indicates a better model fit, with SRMR < 0.1 is indicative of acceptable fit, and SRMR < 0.05 is indicative of excellent fit [19]. A higher CFI indicates better model fit. A CFI > 0.9 is indicative of a good fit, and a CFI > 0.95 is indicative of excellent fit [19]. As opposed to regression modeling, where significant statistics represent the strength of association between the predictors in the response, genomic SEM, a significant *χ*^2^ statistic represents a lack of fit between the observed genetic covariance matrix and the covariance matrix implied by the model [19]. However, with large sample sizes, the *χ*^2^ statistics can be significant regardless of the model, which is not informative. We found all *χ*^2^ statistics were highly significant (p ~ 0), and therefore not reported.

The top-performing factor models in the CFA were further optimized by successive removal of non-significant factor loadings, which is considered standard practice [41]. We additionally fit a model as part of the CFA step to account for the observed correlation between the factors. Specifically, we fit a bifactor model where a “total” CT or SA factor was added, which loaded on all regions, a second bifactor+ model where a total CT or SA factor loaded on all regions and the GWAS results from the corresponding average CT or total SA GWAS from Grasby et al. [18], and a multi-level model where all EFA factors loaded onto a 2nd order factor. The bifactor and bifactor+ models failed to converge and the multilevel models failed to improve model fit in all cases; hence these results are not reported.

### GIBN overlap with alternate networks and parcellations

To explore the possible relevance of GIBNs to other parcellations of the cortex, we used Dice’s Coefficient to measure percent volume overlap. We used permutation testing to determine the significance for each Dice’s coefficient by estimating the probability that the magnitude of overlap occurred by chance. We used 1000 iterations of populating a given network with randomly selected brain regions, calculating its Dice’s coefficient relative to the parcellation of interest, and then comparing the GIBNs true Dice’s coefficient to the null distribution of 1000 Dice’s coefficients. The relative position of Dice’s coefficient for a particular GIBN-to-parcellation comparison within the probability distribution provided the significance. False Discovery Rate (FDR) was used to correct for multiple testing with GIBNs, receptors, networks, and clusters.

First, we conducted a quantitative analysis of the overlap between GIBN’s and 7 canonical RSNs reported by Yeo and colleagues [42]. We quantitatively analyzed the overlap between GIBNs and networks based on 20 neuroreceptor density maps of Hansen et al. [43]. We calculated Dice’s coefficient between each the 4 CT and 6 SA GIBNs and both high and low neuroreceptor densities defined by the top 20%, and bottom 20% receptor densities for serotonin-1a (5-HT1a), serotonin-1b (5HT-1b), serotonin-2a (5-HT2a), serotonin-4 (5-HT4), serotonin-6 (5-HT6), serotonin transporter (5-HTT), alpha-4 beta-2 nicotinic (α4β2), cannabinoid type-1 (CB1), dopamine D1 (D1), dopamine D2 (D2), dopamine transporter (DAT), fluorodopa (fDOPA), gamma-aminobutyric acid A (GABAa), histamine type-3 (H3), muscarinic acetylcholine (M1), metabotropic glutamate receptor-5 (mGluR5), opioid (MOR), norepinephrine (NorEpi), N-methyl-D-aspartic acid (NMDA), vesicular acetylcholine transporter (VachT).

### Multivariate GWAS analysis

Using the GIBNs from our best-fitting model, we used gSEM to generate a multivariate GWAS of each GIBN. The GenomicSEM package *sumstats* program was used to perform the GWAS, with options set for a linear model (continuous outcome) and default parameters for the ‘info’ and ‘maf’ filters (info ≤ 0.6, MAF ≤ 0.01). MAF was determined based on 1000 G Phase3 EUR reference panel. The GWS associations (*p* < 5 × 10^−^^8^) for each GIBN were compared to the significant SNPs reported by Grasby et al. with and without the global correction. The FUnctional Mapping and Annotations (FUMA) package [44] was used to annotate results from each GIBN GWAS, including annotating SNPs to specific genes, identifying independent loci, and identifying potential functional variants. FUMA was run using LD in the 1000 G Phase3 EUR reference panel and the default FUMA parameters.

While CT and SA were examined separately, both for computational limitations and conceptual reasons, we used the multivariate GWAS results to estimate the genetic correlation between CT GIBNs and SA GIBNs, hypothesizing that they would be consistent with the negative genetic correlation between average CT and total SA [16]. Additionally, to examine the degree to which the CT and SA GIBNs genetically resembled the overall CT and SA measures, we estimated the genetic correlation between each GIBN and the average CT and total SA (uncorrected for ICV) as reported in the Grasby et al.

### Polygenicity analysis

We examined the significant SNPs from the GIBN GWAS, as well as SNPs in LD using FUMA to test for functional associations with established behavioral traits and major neuropsychiatric disorders. First, we examined whether observed variants from the GWAS recapitulated GWS SNPs from previous GWASs of neuroimaging traits including cortical GWASs and other structural neuroimaging parameters [17, 45–50]. We also looked for SNPs that were significant in GWASs of 12 neuropsychiatric disorders from the Psychiatric Genomics Consortium (PGC): ADHD [51], alcohol dependence [52], anorexia nervosa [53], autism spectrum disorder [54], bipolar [55], cannabis use [56], MDD [57], obsessive-compulsive disorder (OCD) [58] posttraumatic stress disorder (PTSD) [59], schizophrenia [60], Tourette’s syndrome [61], and anxiety [62]. Finally, FUMA was used to functionally annotate loci that overlapped with previously published GWAS results.

### Genetic correlation with psychopathology

We used cross-trait LDSC to identify links between psychiatric disorders and CT-derived GIBNs as well as psychiatric disorders and SA-derived GIBNs [63]. We estimated the genetic correlation between CT- and SA-derived GIBNs and neuropsychiatric disorders using their GWAS summary statistics [63]. To limit our need for a multiple testing correction, we limited our analyses to the 12 neuropsychiatric disorders noted above. A false discovery rate corrected p value (P_FDR_) was used to correct for the number of GIBNs [10] and disorders [12].

## Results

### Model fit

**Fig. 1:**
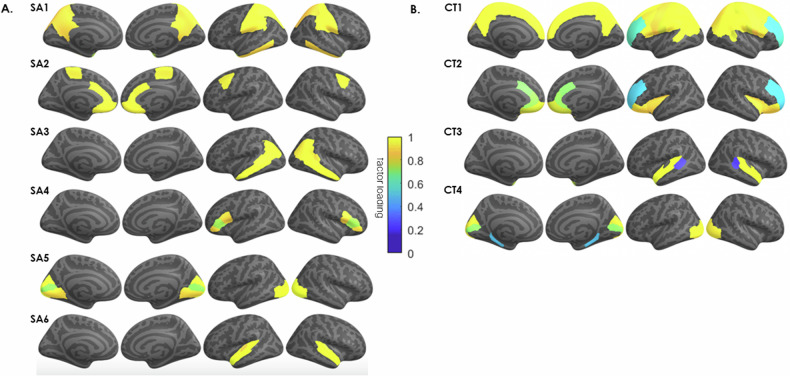
Genetically Informed Brain Networks (GIBNs). Genomic structural equation modeling (gSEM) jointly modeled the genetic architecture of (**A**) cortical surface area (SA), and (**B**) cortical thickness, for 34 brain regions based on GWAS results of Grasby et al. [18]. The model generated 6 *genetically informed brain networks* (GIBNs) from SA phenotype measures. The color overlay on cortical regions represents the magnitude of the factor loadings indicated in the color gradient (yellow = high; blue = low). Subsequent GWAS identified several genome-wide significant hits (*p* < 5 × 10^−8^) associated with each GIBN.

The SA-derived 6-GIBN solution resulted in the best overall model fit to the genetic covariances generated from the GWAS summary statistics (AIC = 22,712,604, CFI = 0.920, SRMR = 0.062). See Supplementary Table S1 for fit statistics for each evaluated model. The 6 SA-derived GIBNs (SA1-SA6) loaded on 24 of the 34 brain regions [18]. The standardized estimates for the 6 SA-derived GIBN models (standardized factor loadings) are presented in Supplementary Table S2 and Fig. 1A. The GIBNs generally encompass contiguous brain regions and many correspond to known neuroanatomical features. SA1 contains loadings for inferior temporal, isthmus cingulate, postcentral, precuneus, superior parietal, supramarginal, and temporal pole. SA2 contains loadings for caudal anterior cingulate, caudal middle frontal, medial orbitofrontal, paracentral, and rostral anterior cingulate. SA3 contains loadings for banks superior temporal sulcus (STS), inferior parietal, and middle temporal. SA4 contains loadings for pars opercularis, pars orbitalis, and pars triangularis, SA5 contains loadings for cuneus, lateral occipital, lingual, and pericalcarine, and SA6 corresponds to the auditory cortex. The 6-factor model indicated substantial correlation between GIBNs (r_g_ = 0.61 to 0.91) as reported in Supplementary Table S3.

The CT-derived 4-GIBN solution resulted in the best model fit (AIC = 17761928, CFI = 0.932, SRMR = 0.077; Supplementary Table S4). Significant non-zero loadings for CT-derived GIBNS loaded on 25 of the 34 brain regions from Grasby et al. See Supplementary Table S5 for the estimated loadings that are visualized in Fig. 1B. As observed with SA models, the CT-derived GIBNs generally encompassed contiguous cortical regions. CT1 contains loadings for banks STS, caudal middle frontal, inferior parietal, paracentral, pars opercularis, post-central, pre-central, precuneus, rostral middle frontal, superior frontal, superior parietal, and supramarginal cortices. CT2 contains loadings for the caudal anterior cingulate, frontal pole, insula, lateral orbitofrontal, medial orbitofrontal, pars orbitalis, rostral anterior cingulate, and rostral middle frontal. CT3 contains loadings for banks STS, superior temporal, and temporal pole. CT4 contains loadings for cuneus, lateral occipital, parahippocampal, and pericalcarine cortices. The CT-derived GIBNs were moderately to highly correlated (*r*_g_ = 0.67 to 0.87; Supplementary Table S6).

**Fig. 2:**
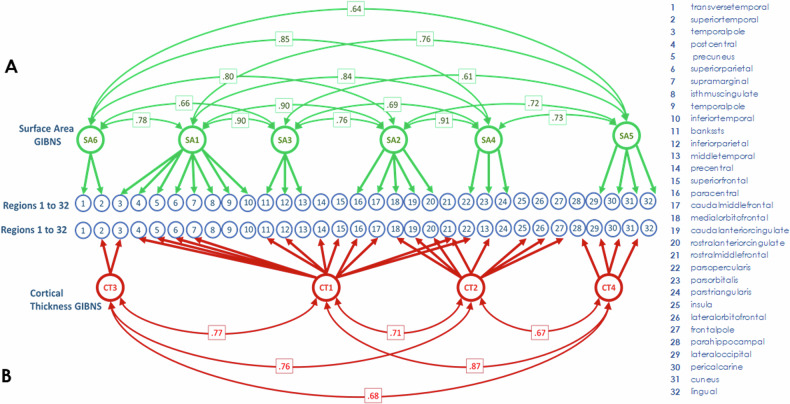
Graph of genomic structural equation modeling (gSEM) results. The blue circles, numbered from 1 to 32, represent the cortical surface area (SA) and cortical thickness (CT) of regions defined by the Desikan–Killiany atlas in the figure legend. Two separate gSEMs were carried out on **A** Latent SA variables, indicated by green circles, representing the genetic contributions from regional SA, which are specified by thick green lines and arrows. Thin green lines connect genetically related SA variables with their genetic correlation strength (*r*_g_) indicated in green boxes. **B** Latent CT variables, indicated by red circles, represent the genetic contributions from regional CT, which are specified by thick red lines and arrows. Thin red lines connect genetically related CT variables with their genetic correlation strength (*r*_g_) indicated in red boxes. SA surface area, CT cortical thickness, GIBN genetically informed brain network.

Factor diagrams for SA- and CT-derived GIBNs are presented in Fig. 2. Consistent with prior work, the SA-derived GIBNs were largely distinct from CT-derived GIBNs, although some regional overlap exists. For example, SA5 and CT4 are both 4-region GIBNs, with 3 overlapping regions.

We then performed a sensitivity analysis by switching the order of the chromosomes, with the even chromosomes used in the EFA and the odd chromosomes used in the CFA. See Supplementary Tables S7–S12 for fit statistics and model estimates. The best fitting model from this sensitivity analyses had seven SA GIBNs and four CT GIBNs, with very similar, but slightly worse CFI fit statistics than the model described above (SA CFI = 0.89 and SRMR = 0.064, vs CFI of 0.92 and SRMR of 0.62 for the original SA GIBNs; CT CFI = 0.86 and SRMR = 0.077 vs CFI of 0.93, SRMR = 0.077 for the original CT GIBNs; AIC are not comparable). Interestingly, even though the fit was similar, the factor structure differed substantially from our original runs, although there were points of correspondence. For example, SA factor 2 from the 7-factor sensitivity analysis model corresponds to SA5 of the original model. The CT1 and CT2 GIBNs from the original runs were subsets of larger regions identified as CT1 and CT2 in the sensitivity analysis. We decided to continue the investigation of the SA and CT GIBNs from our original models based on CFI and SRMR, but we note that the sensitivity analyses indicate other solutions may be viable alternatives to this factor structure.

### Overlap of SA GIBNs and twin-derived genetic SA parcellations

**Fig. 3:**
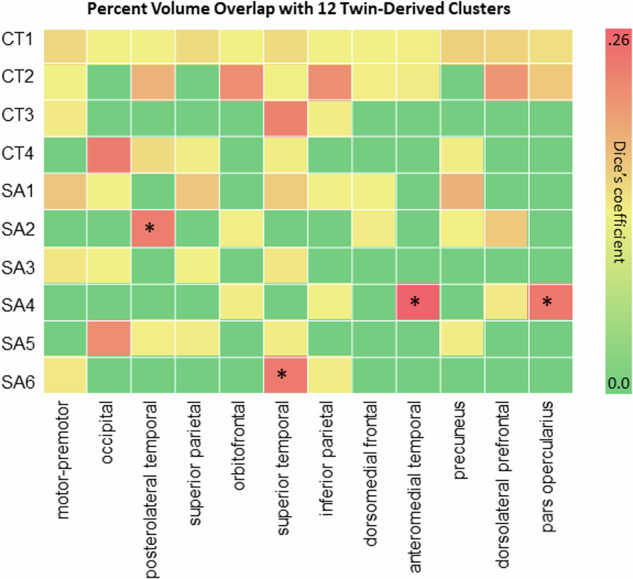
Genetic parcellation of twin brains. We examined the percent volume overlap as measured by Dice’s coefficients for the overlap between 6 SA GIBNs and the 12 clusters reported by Chen et al. [11] derived from twin brain data. *FDR-corrected *p* values for percent volume overlap with 12 clusters are indicated by an asterisk. SA surface area, CT cortical thickness, GIBN genetically informed brain network.

We computed Dice’s coefficients (DCs) and corresponding *p* values between the SA GIBNs and the 12 SA correlation networks reported by Chen et al. [11]. Our results (Fig. 3) showed a high overlap between SA2 and the posterolateral temporal (DC = 0.205, p_FDR_ = 0.02), SA4 and pars opercularis (DC = 0.219, p_FDR_ = 0.02), SA4 and anteromedial temporal network (DC = 0.256, p_FDR_ = 0.02), SA4 and pars opercularis network (DC = 0.219, p_FDR_ = 0.02), SA4 and anteromedial temporal network (DC = 0.259, p_FDR_ = 0.02), and SA6 and superior temporal network (DC = 0.211, p_FDR_ = 0.02).

### Overlap of GIBNs and RSNs

**Fig. 4:**
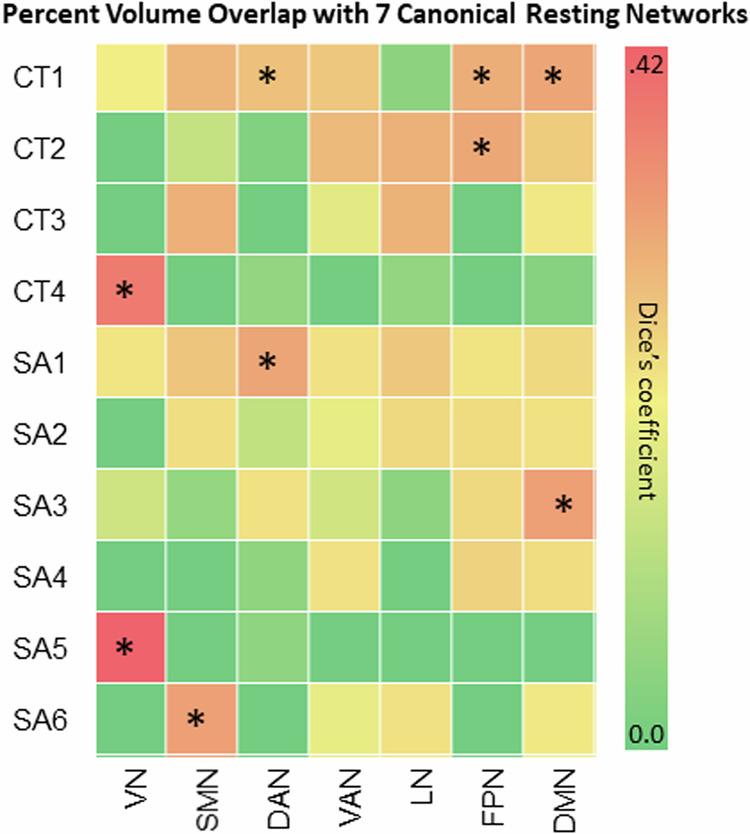
Canonical resting-state networks. Percent volume overlap (Dice’s coefficients) between GIBNs (4 CT and 6 SA) and canonical resting-state networks for the 7-network parcellation scheme by Yeo and colleagues (2011). *FDR-corrected *p* values for percent volume overlap between GIBNs and 7 canonical resting-state networks. VN visual network, SMN somatomotor network, DAN dorsal attention network, VAN ventral attention network, LN limbic network, FPN frontoparietal network, DMN default mode network, SA surface area, CT cortical thickness, GIBN genetically informed brain network.

The Dice’s coefficients and corresponding *p* values of GIBNs and the Yeo et al. [42] 7 RSNs (Fig. 4) showed that CT4 and SA5 had relatively high overlap with the visual network (CT4:DC = 0.353, p_FDR_ = 0.008; SA5:DC = 0.424, p_FDR_ = 0.008), while CT1 and SA3 had high overlap with the DMN (CT1:DC = 0.232, p_FDR_ = 0.008, SA3:DC = 0.249, p_FDR_ = .008, CT1 and SA1 with DAN (CT1:DC = 0.159, p_FDR_ = 0.008; SA1:DC = 0.232, *p* = 0.008), CT1 and CT2 with FPN (CT1:DC = 0.210, p_FDR_ = 0.008; CT2:DC = 0.225, p_FDR_ = 0.008), and SA6 with SMN (DC = 0.247, p_FDR_ = 0.008.

### GIBN overlap with high/low neuroreceptor density regions

**Fig. 5:**
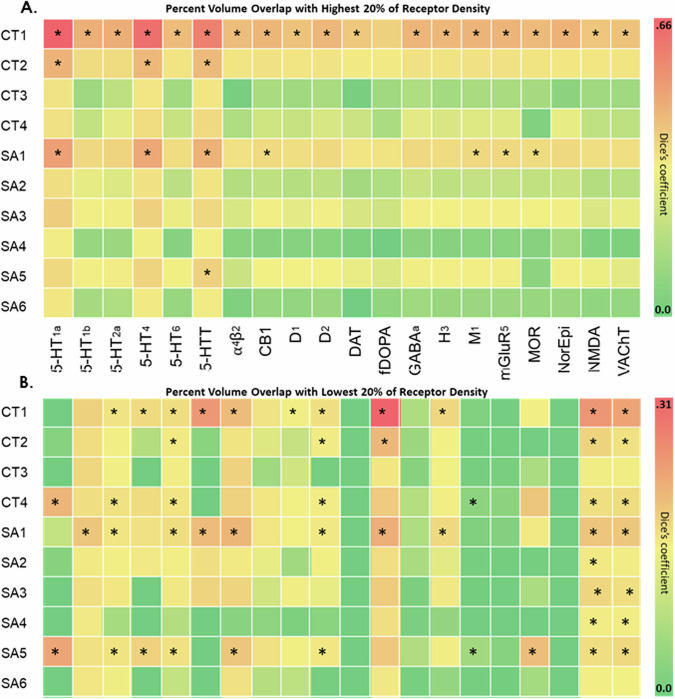
Receptor density. Dice’s coefficients and corresponding *p* values between each of the 4 CT and 6 SA GIBNs and neuroreceptor density. The 20 receptors included serotonin 1a (5-HT1a), serotonin 1b (5-HT1b), serotonin 2a (5-HT2a), serotonin 4 (5-HT4), serotonin 6 (5-HT6), serotonin transporter (5-HTT), alpha-4 beta-2 nicotinic (α4β2), cannabinoid type 1 (CB1), dopamine D1 (D1), dopamine D2 (D2), dopamine transporter (DAT), fluorodopa (fDOPA), gamma aminobutyric acid A (GABAa), histamine type 3 (H3), muscarinic acetylcholine (M1), metabotropic glutamate receptor 5 (mGluR5), opioid (MOR), norepinephrine (NorEpi), N-methyl-D-aspartic acid (NMDA), vesicular acetylcholine transporter (VAchT). In Hansen et al. [43] and the 10 GIBNs maps were used to calculate Dice’s coefficient and corresponding FDR-corrected *p* values for **A** the highest 20% receptor density, and **B** the lowest 20% receptor density (* FDR-corrected *p* value < 0.05). The results, consisting of 200 Dice’s coefficients (20 receptors × 10 GIBNs), are displayed in heatmaps. SA surface area, CT cortical thickness, GIBN genetically informed brain network.

We examined the overlap between CT and SA GIBNs and regions of highest (top 20%; Fig. 5A) and lowest (bottom 20%, Fig. 5B) neuroreceptor densities. We found that CT1 overlapped a region of high neuroreceptor densities for many types of neuroreceptors and a region of low fDOPA receptor density. CT2 and SA2 overlapped regions of high 5-HT1a, 5-HT4, and 5HTT receptor density. SA5 overlapped the high 5HTT receptor-density region. (Fig. 5A).

### GWAS of GIBNs

**Fig. 6:**
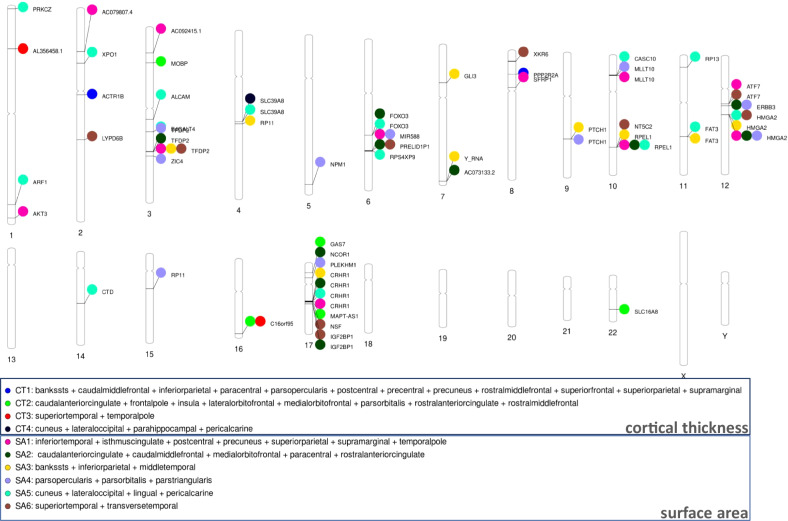
SNPs for GIBNS derived from surface area and cortical thickness. Phenogram of GWS SNPS associated with six genetically informed brain networks (GIBNs) derived from surface area (SA) and four GIBNs derived from the cortical thickness (CT).

To identify specific genetic variants that may be influencing the GIBNs, we performed a multivariate GWAS on each SA- and CT-derived GIBN. Manhattan plots for SA- and CT-derived GIBN GWASs, their associated quantile-quantile (QQ) plots, and genomic inflation factors (λ) are provided in Supplementary Figs. S5–14. We observed moderate *p* value inflation (λ values between 1.06 and 1.16). However, the single-trait LD Score regression intercepts for SA- and CT-derived GIBNs were all less than 1.02, indicating that the apparent inflation was likely due to polygenicity. A total of 5,843 GWS (*p* < 5 × 10^−^^8^) variants were associated with the GIBNs. FUMA [44] mapped these variants to 74 independent regions, including 64 loci associated with the 6 SA-derived GIBNs and 10 loci associated with the 4 CT-derived GIBNs. A phenogram [64] of the genetic associations is presented in Fig. 6. A list of all GWS loci is provided in Table S13. Except for two novel SNPs, all others were previously identified in Grasby et al. [18]. in either the analyses adjusted for global SA/CT or the unadjusted analyses. The first novel SNP, rs3006933, near the genes *SDCCAG8* and *AKT3* on chromosome 1, was associated with SA1 (*p* = 4.08 × 10^−9^). The other novel SNP, rs1004763, on chromosome 22 in the vicinity of the gene *SLC16A8*, was associated with CT2 (*p* = 3.41 × 10^−^^08^). Notably, many of the 75 GWS loci associated with GIBNs were not associated with global measures, but only with individual CT/SA regional measures, and half (37 out of 75) were more significantly associated with the GIBNs than the corresponding global measures. Using FUMA we found no significant enrichment of a particular tissue type in either CT- or SA-derived GIBNs and no enriched expression of developmental genes or regulators.

### Genetic correlation between CT and SA

Although CT and SA regions were analyzed separately, we examined the genetic correlation between CT and SA using LDSC analysis of the GIBN GWAS results. The mean genetic correlation between SA GIBNs and CT GIBNs is −0.22 (−0.43 to −0.08; Table S14), whereas the mean genetic correlation between the 6 SA GIBNs is 0.77 (0.61 to 0.91; Table S3) and the mean genetic correlation between the 4 CT GIBNs is 0.76 (0.71 to 0.87; Table S6). The dramatically lower correlation between CT and SA compared to within SA and compared to within CT GIBNs supports separate gSEM analyses of CT and SA phenotypes.

### LDSC analysis of genetic correlation

We examined the genetic correlation between CT and SA GIBNs and psychiatric disorders. The LDSC analysis of SA GIBNs is reported in Table S15 and Figure S15. ADHD exhibited a significant negative genetic correlation with all SA-derived GIBNs except SA4 (r_g_ = −0.13 to −0.20, *p* = 3.29 × 10^−^^6^ to 0.0038, p_FDR_ = 0.00040 to 0.039). Significant positive genetic correlations were observed between bipolar disorder and SA1, SA2, SA4, and SA5 (r_g_=0.10 to 0.14, *p* = 3.00 × 10^−^^4^ to 0.0047, p_FDR_ = 0.012 to 0.043). Interestingly, we observed significant genetic correlations between MDD and SA-derived GIBNs, but in the opposite direction as bipolar disorder. We found a significant negative correlation between MDD and SA6, which was not associated with bipolar disorder (r_g_ = −0.10, *p* = 0.0011, p_FDR_ = 0.17). Negative correlations were non-significant after multiple-testing correction between MDD SA1-SA3, and SA5 (r_g_ = −0.057 to −0.080, p_unc_ = 0.0090 to 0.046), while SA4 was not genetically correlated with MDD (*p* = 0.12). SA4 was significantly correlated with cannabis use disorder (r_g_=0.15, *p* = 4.00 × 10^−^^4^, p_FDR_ = 0.012), while SA2 correlation with cannabis use was non-significant (r_g_=0.11, p_unc_ = 0.011).

Fewer genetic correlations were significant between CT-derived GIBN regions and psychiatric disorders (Table S16 and Figure S16). CT3 and CT4 were negatively correlated with alcohol use disorder, exhibiting the strongest correlations with any traits that we examined (CT3 r_g_ = −0.35, *p* = 3 × 10^−^^4^, p_FDR_ = 0.012; CT4 r_g_ = −0.31, *p* = 7 × 10^−^^4^, p_FDR_ = 0.014). We found a negative nominally significant correlation between alcohol use disorder and CT1 (r_g_ = −0.18, p_unc_ = 0.035, p_FDR_ = 0.22). CT3 had a positive nominally significant correlation with OCD (r_g_=0.22, p_unc_ = 0.0091, p_FDR_ = 0.078).

The global measures for thickness and SA are genetically correlated to many of the same psychopathology traits as the GIBNs, but the genetic correlations with global measures are almost always less significant than the genetic correlations with the most strongly associated GIBNs. The details of these results are provided in Tables S15 and S16.

## Discussion

The goal of the present study was to leverage the pleiotropic architecture of the human cortex to investigate genetic factors underlying CT and SA, and to identify further links between the genetics of CT, SA, and psychopathology. We applied gSEM to jointly model the genetic architecture of 34 brain regions using results from the ENIGMA-3 GWAS [18]. The process was undertaken with gSEM to generate several possible solutions, from which the best-model fit was selected. This solution organized brain regions to optimally assign genetic pleiotropy to 6 SA- and 4 CT-derived latent factors, which we have termed *genetically informed brain networks* (GIBNs).

The GIBNs we generated may be compared to similar structures generated from twin studies. Using 400 twin pairs, Chen et al. generated twelve genetically-informed clusters from vertex-based SA measures [11]. Chen et al. [11]. reported heritability estimates and genetic correlations between genetically informed parcels that are more consistent with classical anatomically-defined sulcal and gyral boundaries, Brodmann definitions, and cytoarchitectural patterns than our GIBNs. The slight differences in model fit between potential alternating models from our main analyses and sensitivity analyses indicate that this is one of several viable solutions with similar fit statistics. Even so, it is instructive to examine ways this solution corresponds to prior parcellations of the cortex based on genetic correlation as determined in twin studies as well as neurobiological and functional parcellations.

The best-fitting SA-derived GIBNs overlap with several canonical RSNs, such as visual network and SA5 (Dice’s coefficient=0.424), which is composed of cuneus, lateral occipital, lingual, and pericalcarine cortices [42]. Twin-based non-linear multidimensional heritability estimates are among the highest for the visual network (left h^2^_m_ = 0.53; right h^2^_m_ = 0.45) and auditory network (left h^2^_m_ = 0.44; right h^2^_m_ = 0.60) [65]. SA6, which includes superior and transverse temporal cortices, overlaps the auditory cortex from twin-derived genetic parcels (Dice’s coefficient = 0.211; Fig. 3). The functional specializations of the human auditory cortex [42], which include parts of the lateral prefrontal cortex, Broca’s area, and subcentral regions, are needed for human vocalization and language [66, 67]. The dorsal attention network (DAN), which directs voluntary allocation of attention, has substantial overlap with SA1 (Dice’s coefficient = 0.232, Fig. 4) that is comprised of superior parietal, supramarginal, postcentral, precuneus, isthmus cingulate, and inferior temporal regions. A noteworthy omission from SA1, an important feature of the DAN, are the frontal eye fields (FEF) [68]. Since FEF is not a FreeSurfer parcellation output, it may be poorly represented in the ENIGMA cortical GWAS. The DAN has relatively high twin heritability estimates (left h^2^ = 0.45; right h^2^ = 0.40) [65]. SA4 partially overlaps (Dice’s coefficient=0.259) the frontoparietal network (FPN), which includes pars opercularis, pars orbitalis, and pars triangularis, but lacks the critical temporoparietal structures [69] (Fig. 4). One advantage of using gSEM and identifying GIBNS as a strategy is that it allows us to align the genetic correlation of clusters of regions with neurobiological features such as connectivity and gene expression, and implicates specific GIBN-associated genetic variants and the degree to which they align with other traits such as psychopathology.

The overlap of GIBN GWS loci with prior GWAS of neuroimaging phenotypes or psychiatric disorders firmly points to the relevance of GIBN-related (multi-regional SA and CT) variants to brain structure and cognition. First, we note that novel variant rs3006933 has been previously associated with subcortical volumes [70]. Novel variants rs3006933 and rs1004763 [17, 48] have been associated with neuroimaging phenotypes of corpus callosum white matter microstructure [71]. A comparison of our GIBN GWAS with published psychiatric disorder GWAS results found that multiple SNPs linked to SA-derived GIBNs were also implicated in a GWAS of schizophrenia [60]. Specifically, we identified a cluster of 4 loci in the *CRHR1* gene strongly associated with SA-derived GIBNs (rs62057153 associated with SA1) in our GWAS (*p* = 5.22 × 10^−17^ to 8.45 × 10^−21^). We also observed an association between CT1 and rs11692435 (*p* = 1.17 × 10^−12^), a schizophrenia-related locus, within the *ACTR1B* gene. Finally, CT- and SA-derived GIBNs were associated with schizophrenia risk variants in the *SLC39A8* gene; namely rs13107325 was associated with CT5 and rs13135092 was associated with SA5. No other traits had GWS variants associated with any of the GIBNs.

Many GIBN-associated SNPs have been associated with other cognitive, behavioral, neuroanatomical, neurofunctional, and neuropsychiatric phenotypes. In addition to rs3006933 noted above [16], SA6-linked locus rs9909861 [72] and SA5-linked SNP rs7570830 [16] have been associated with subcortical volumes. Multiple loci associated with SA-derived GIBNs that encompass temporal, parietal, and temporo-parietal association cortices, including the SA1-linked locus rs10109434 [73], the SA3-linked SNP rs2299148 [74], and the SA6-linked locus rs9909861 [74–78] have been implicated in academic attainment and cognitive ability. Regions in SA6, namely superior temporal gyrus, and SA3, namely supramarginal gyrus were the most strongly linked to academic attainment in the UKB sample [79]. The same regions were reported independently in the Queensland Twin Imaging and HCP samples [80]. The SA5-linked locus rs6701689 has been reported for risk tolerance [81]. However, a role for SA5 in risk tolerance is unsupported. Risk tolerance is linked to cerebellar, midbrain, and prefrontal cortical anatomy, as well as glutamatergic and GABAergic neurotransmission [81, 82]. The CT4-associated locus rs13107325 has been associated with many traits including schizophrenia [83–90], bipolar disorder [87, 88], Parkinson’s disease [89, 90], sedentary behavior [70, 91] and risk taking [81], as well as cognition, intelligence, and educational attainment [74–78, 92]. CT4 includes the parahippocampal and fusiform gyri, which have firmly established links to schizophrenia [93] and sedentary behavior [94].

Behavioral traits and neuropsychiatric disorders showed distinct genetic correlations with SA-derived GIBNs that differ markedly from correlations with CT-derived GIBNs. CT3, located in the middle and superior temporal cortices, and CT4, located in the visual perceptual cortex, were strongly negatively correlated with alcohol use disorder. This divergent relationship between CT-derived and SA-derived networks is consistent with the ENIGMA-3 cortical GWAS where a similar pattern of positive and negative correlations between total brain SA and behavioral traits/disorders was found, but average CT correlations with behavioral traits/disorders were non-significant [18]. Specifically, the ENIGMA-3 GWAS found that total SA was significantly positively correlated with cognitive function, educational attainment, Parkinson’s disease, and anorexia nervosa, but significantly negatively correlated with MDD, ADHD, depressive symptoms, neuroticism, and insomnia. In addition, the SA-derived GIBNs showed distinct genetic relationships to several psychiatric disorders. Several SA-derived GIBNs (SA1, SA2, SA4, SA5) were positively correlated with bipolar disorder, whereas SA-derived GIBNs (SA1, SA2, SA3, SA5, SA6) were negatively correlated with MDD, buttressing prior evidence that MDD and Bipolar are distinct conditions with diverging genetics [27]. While the relationship between these SA-derived GIBNs and MDD converge with the findings from the ENIGMA total SA results, the relationship with bipolar disorder was novel. Thus, GIBNs may provide additional power to detect genetic relationships when their strength across cortical regions is heterogenous.

Interestingly, although several GIBN-associated SNPs were associated with schizophrenia, no GIBNs were significantly genetically correlated with schizophrenia (r_g_=0.029 to 0.034; *p* values > 0.30). While this may be counterintuitive, genetic correlation between phenotypes predicts an overlap in SNPs, but the reverse may not be true. A genetic correlation could be zero when many variants affect both traits, but the direction of effects are uncorrelated across variants [95].

There is ample evidence that genetic variants that influence SA are distinct from genetic variants that influence CT [18]. The results of Panizzon et al. [29] and Grasby et al. [18] compared to van der Meer et al. [16] are focused on different, albeit related measures. van der Meer et al. [16] primarily focused on the overlap of individual genetic variants associated with CT and SA and only secondarily on their genetic correlation. By contrast, Panizzon et al. [29] focused on genetic correlation from twin data, which means genetic marker associations were not available. Grasby et al. [18] was focused primarily on GWAS results and secondarily on reporting genetic correlations between CT and SA. Indeed, the results of van der Meer [16] are completely consistent with the results of Grasby et al. [18], with the former reporting a genetic correlation between SA and CT of – 0.26, and the latter of – 0.32. However, van der Meer et al. [16] reports that the 4016 out of 7941 causal variants are shared between CT and SA. Therefore, the relatively high genetic overlap, but low genetic correlation, is due to a mixture of opposing and agreeing effects from variants. Genetic variation affecting gene regulation in progenitor cell types, present in fetal development, affects adult cortical SA [96]. An increase in proliferative divisions of neural progenitor cells leads to an expanded pool of progenitors, resulting in increased neuronal production and larger cortical SA, which is more prevalent in gyrencephalic species (e.g. humans, primates) [97]. By contrast, loci near genes implicated in cell differentiation, migration, adhesion, and myelination are associated with CT. Our findings suggest this distinction holds for SA-derived compared to CT-derived GIBNs. We hypothesize that the unique genetic correlations of SA-derived GIBNs and CT-derived GIBNs with behavioral traits/disorders may be explained by the distinct developmental functions of their associated genes [98].

### Limitations

A number of limitations deserve consideration in interpreting the present findings. First, we note that even with the large sample size of the Grasby et al. [17, 18] GWAS, there were many models that fit approximately as well as our final model in terms of the number of GIBNs and the clustering of the regions. Our sensitivity analyses, in which the order of the chromosomes in the EFA and CFA were switched provided alternate solutions. Many of these solutions had similar CFI and SRMR scores, indicating acceptable fit according to the CFI and SRMR criteria, but none had a consistently excellent fit. This may be an artifact of the number of regions examined and the dimensionality of the model. Therefore, our set of GIBNs should be considered a working model rather than the only possible genetic parcellation of the cortex. This model is useful, in that it allows us to generate a set of genetic associations with a smaller number of tests than considering each of the regions individually.

There are also other limitations to consider. We only examined genetic correlation with a handful of traits to reduce the multiple testing burden, but note that there are other traits worthy of interest, but beyond the scope of this manuscript. This also includes traits like insomnia and restless sleep, given the reported genetic relationship between psychiatric disorders and diurnal sleep patterns [32]. Other traits such as Parkinson’s and Alzheimer’s disease would also be of interest [99, 100]. There are other analyses that can be run to further interpret the multivariate GWAS results, including Mendelian Randomization to support a causal relationship between cortical structure and psychiatric disorders and neurological traits. However, given the instability of the model estimates in the sensitivity analyses, and large number of models with similar fit indices, we think it is premature to exhaustively investigate the characteristics of these GIBNs. It is likely the factor structure we have presented will be supplanted in the future as higher spatial resolution genetic associations become available. The present gSEM was based on the GWAS results of Grasby et al. [17, 18], which averaged left and right hemisphere phenotypic measures. Additionally, Grasby et al. examined the 34 cortical regions as defined by the Desikan-Killiany atlas. A high-resolution GWAS of the cortex would allow more flexibility and redefining parcellation boundaries informed by genetic pleiotropy and likely yield GIBNs which correspond more closely to anatomical and functional boundaries. However, simultaneously analyzing GWAS results for each of 100 s or 1000 s of vertices would present a computational challenge without major advances in gSEM methodology. Therefore, the GIBNs we presented here are a proof-of-concept that genetic correlation can be used to enhance the interpretation of high-dimensional GWAS results and provide novel insights into the relationship between neuroimaging phenotypes and psychiatric disorders.

### Conclusion

We harnessed the pervasive pleiotropy of the human cortex to realize a genetically-informed parcellation that is neurobiologically distinct from functional, cytoarchitectural, and other established cortical parcellations, yet harbors meaningful topographic similarities to other network schemas. Strong genetic correlation between GIBNs and several major neuropsychiatric conditions, coupled with clear confirmation that nearly all GIBN-associated SNPs play a role in cognitive, behavioral, neuroanatomical, and neurofunctional phenotypes, begins to expose the deeply interconnected architecture of the human cortex. Applying gSEM to model the joint genetic architecture of complex traits and investigate multivariate genetic links across phenotypes offers a new vantage point for mapping genetically informed cortical networks, although with limitations that must be carefully considered when results are interpreted.

## Supplementary information

SUPPLEMENTAL Materials

## Data Availability

GWAS summary statistics used in this paper are available on the ENIGMA consortium website (http://enigma.ini.usc.edu/research/download-enigma-gwas-results). The Genomic SEM package used to analyze the data is publicly available at https://github.com/GenomicSEM/GenomicSEM. The *ldsc* package is publicly available at https://github.com/bulik/ldsc. The results of the multivariate GWASs of the CT- and SA-derived GIBNs are available at https://pgc-ptsd.com/about/workgroups/imaging-workgroup/. All methods were performed in accordance with relevant guidelines and regulations.
