# Supplementary material for: Genomic structural equation modeling reveals latent phenotypes in the human cortex with distinct genetic architecture
Source: Transl Psychiatry. 2024 Oct 24;14:451. doi: 10.1038/s41398-024-03152-y (PMC11502831; doi:10.1038/s41398-024-03152-y)
Supplement: Supplementary file 1 — SUPPLEMENTAL Materials [file 41398_2024_3152_MOESM1_ESM.docx]

**SUPPLEMENTARY FIGURES
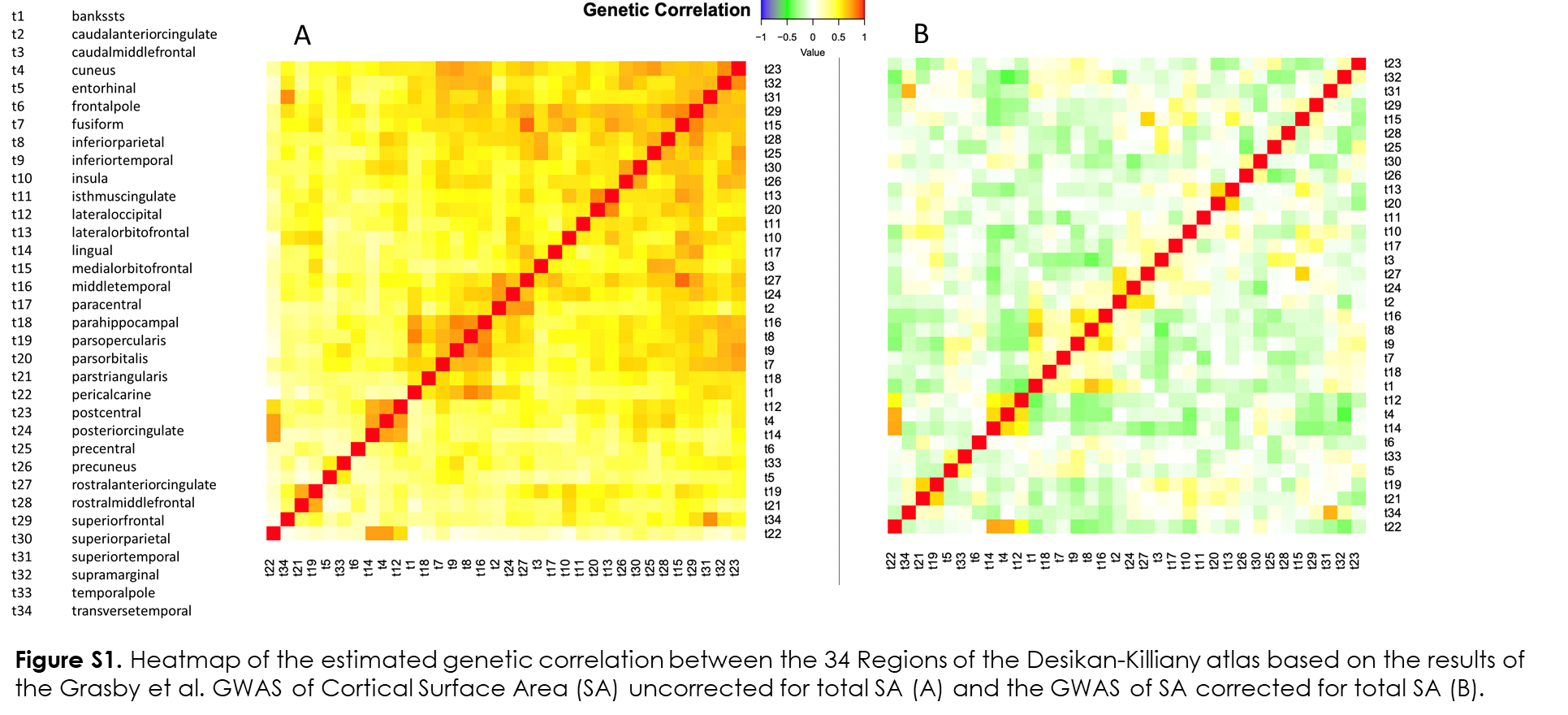
**

**
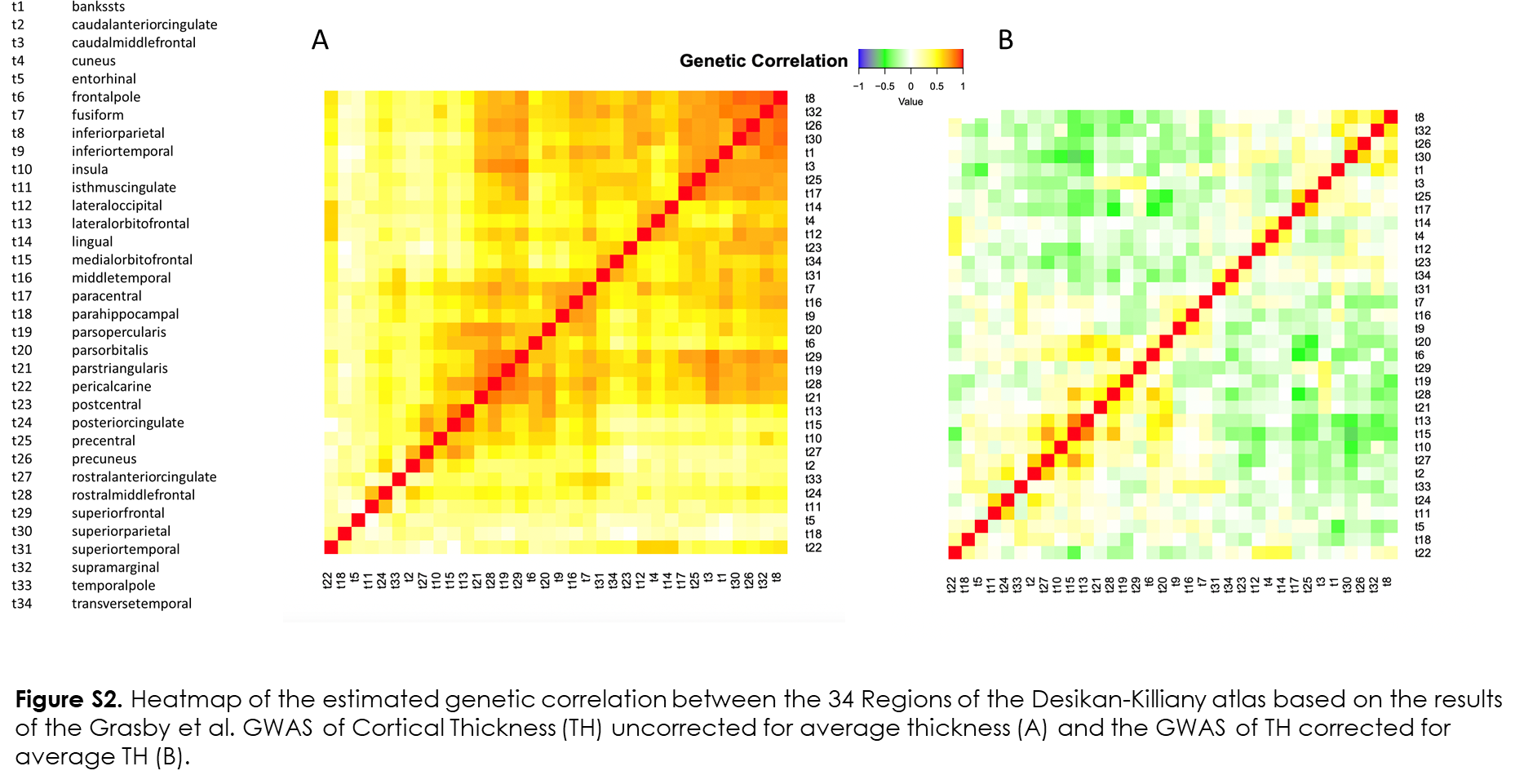
**

**
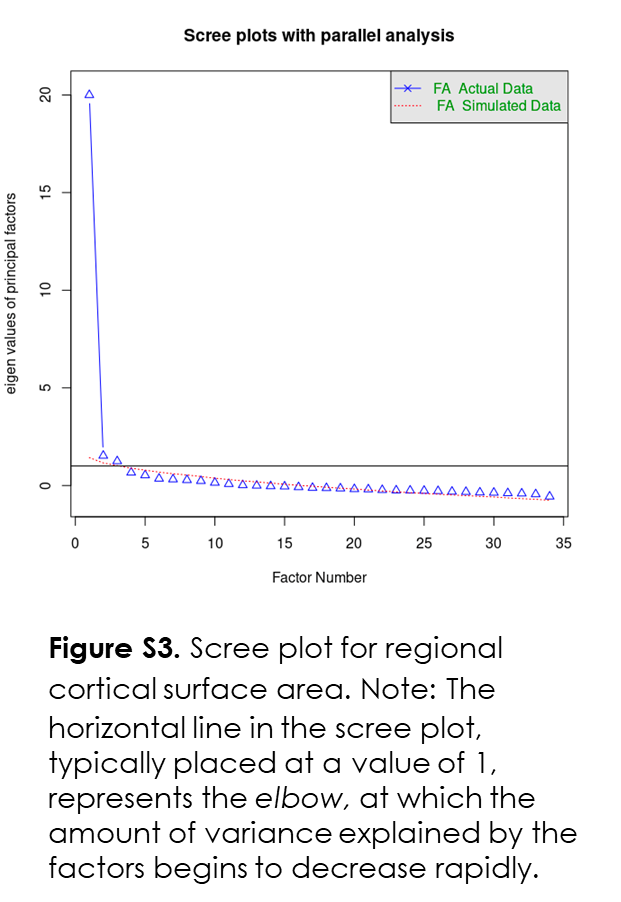
**

**
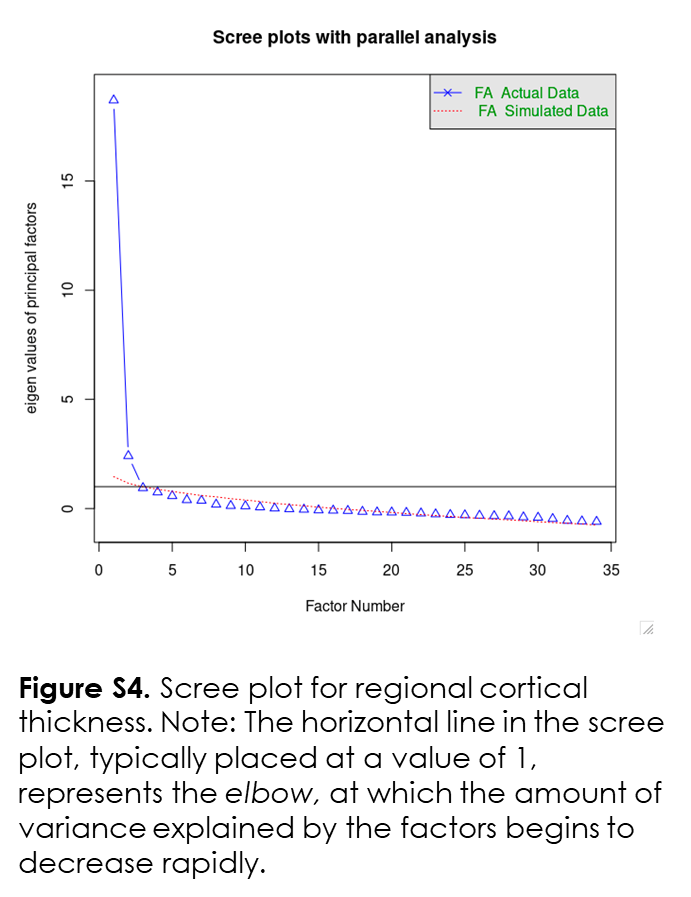
**

**
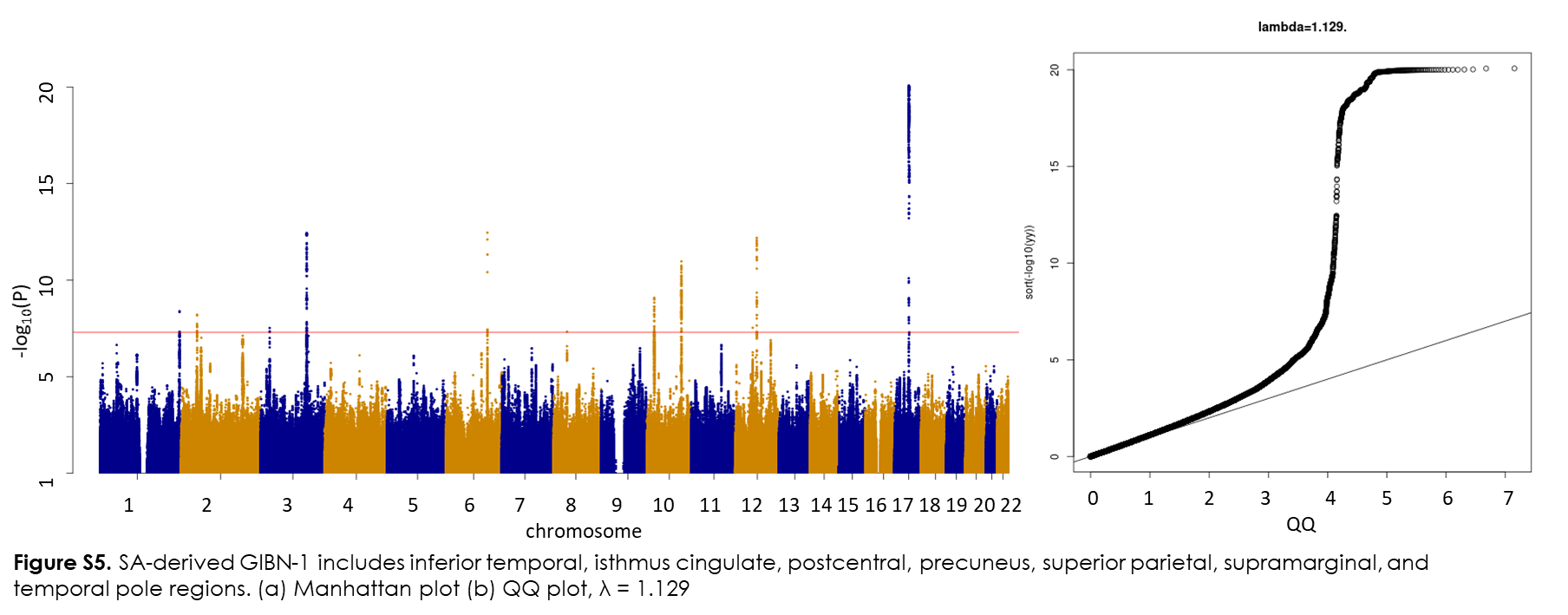
**

**
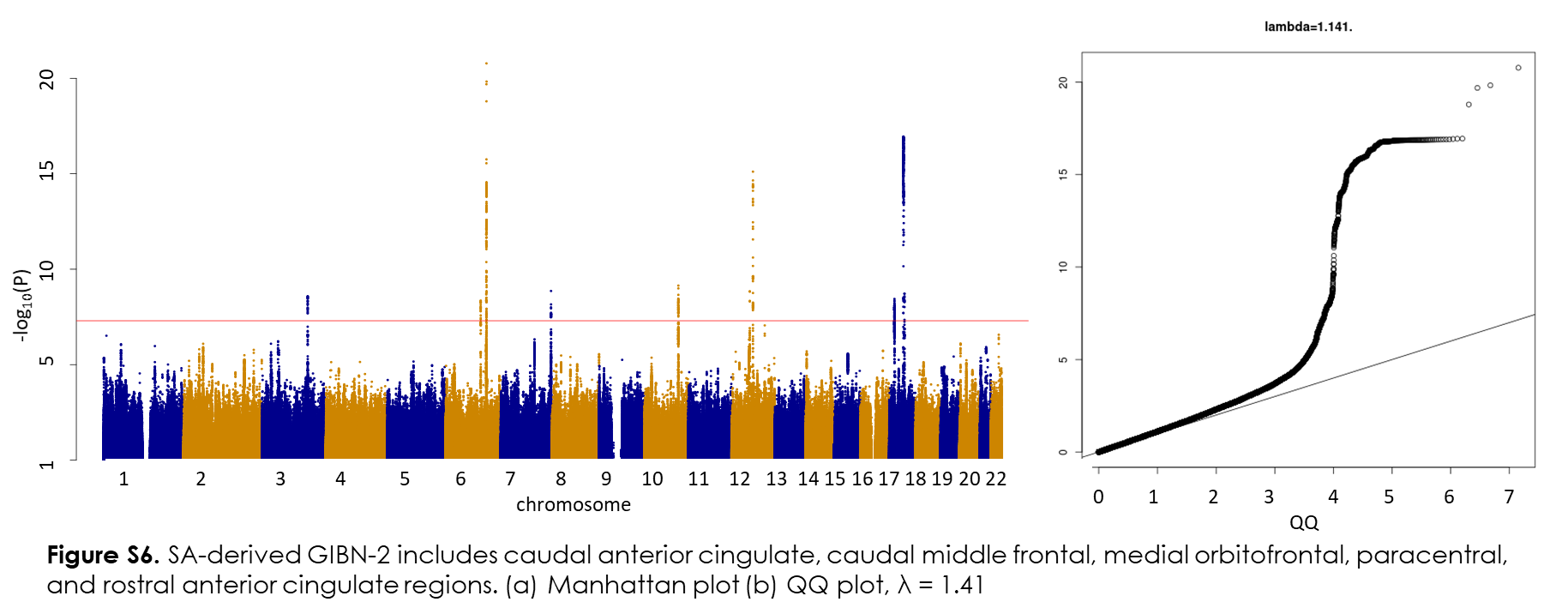
**

**
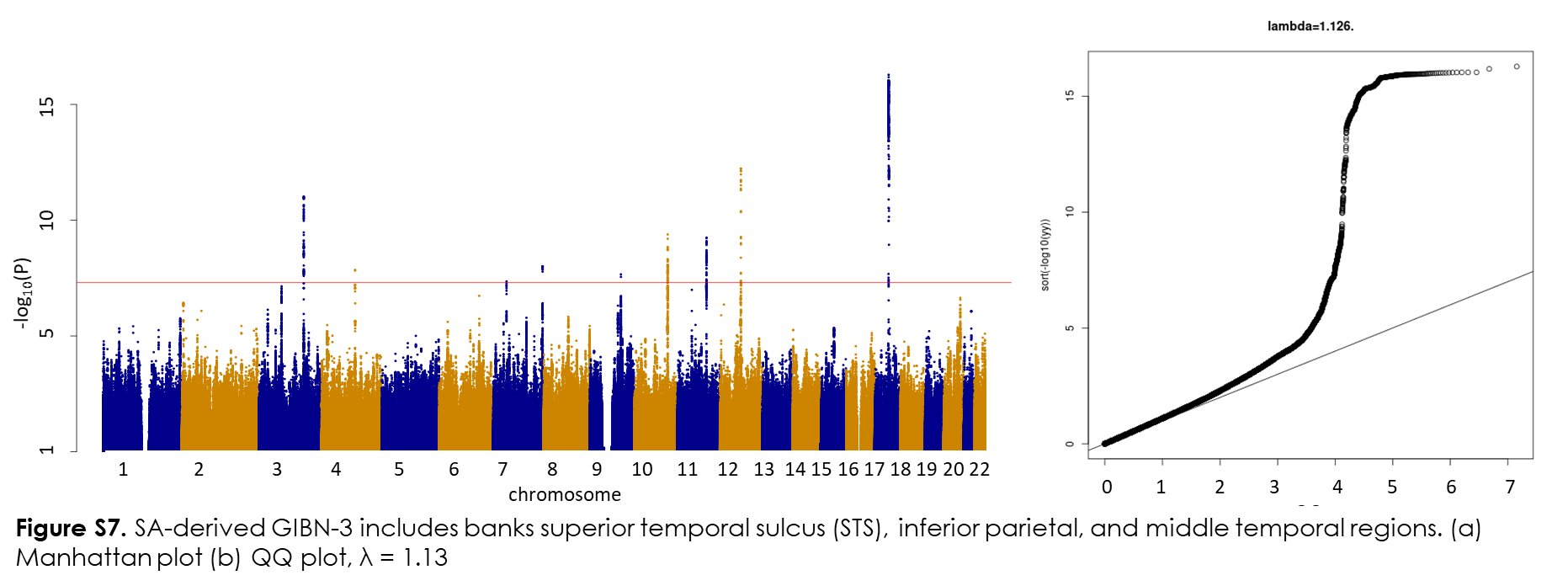

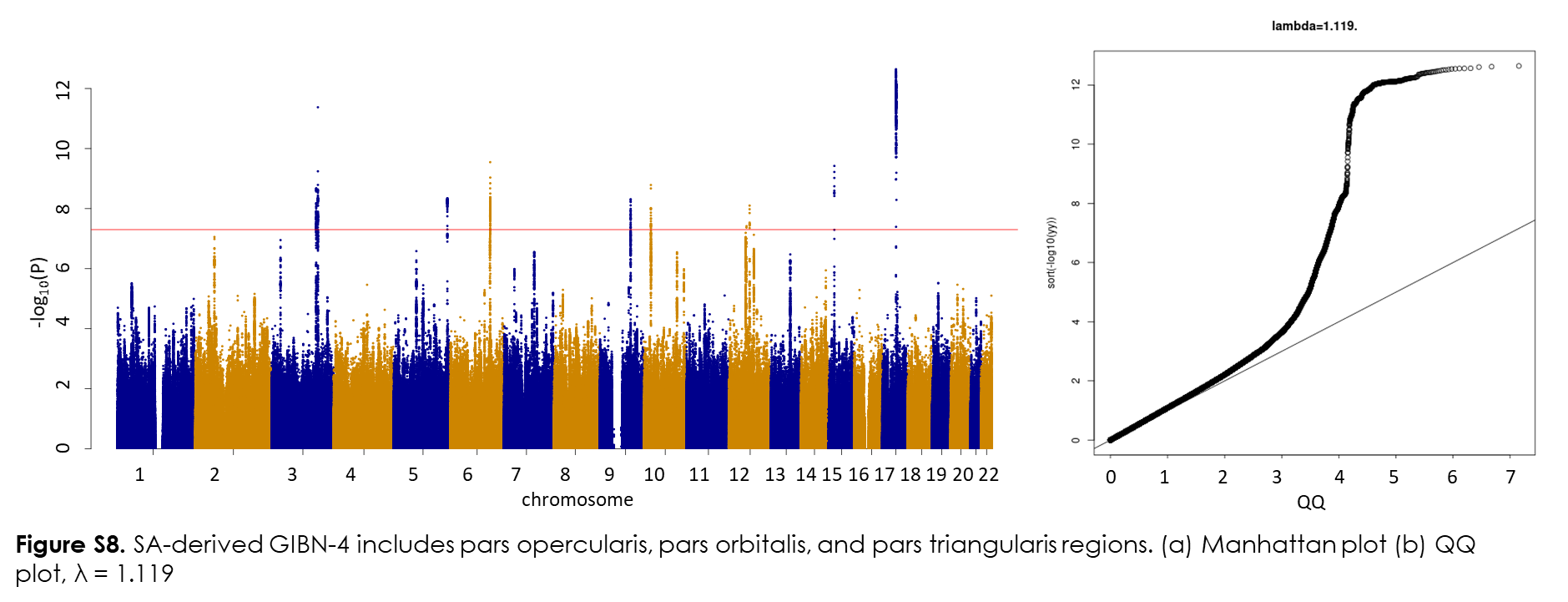
**

**
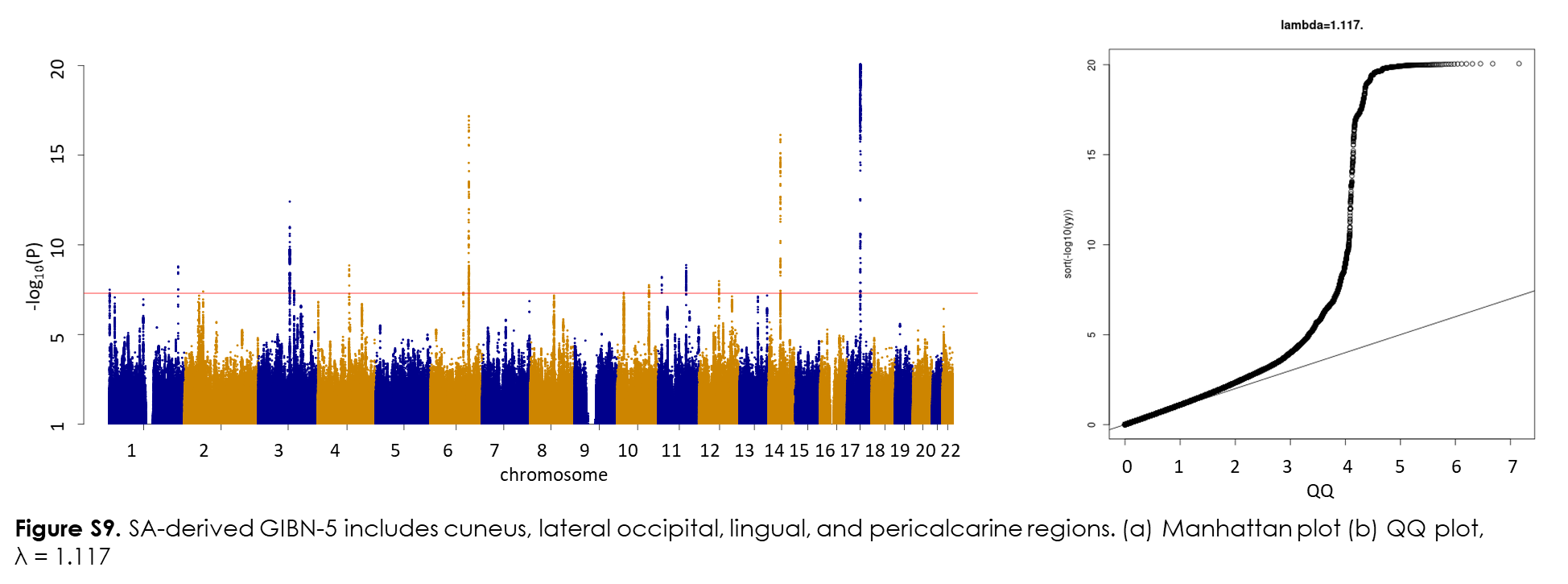
**

**
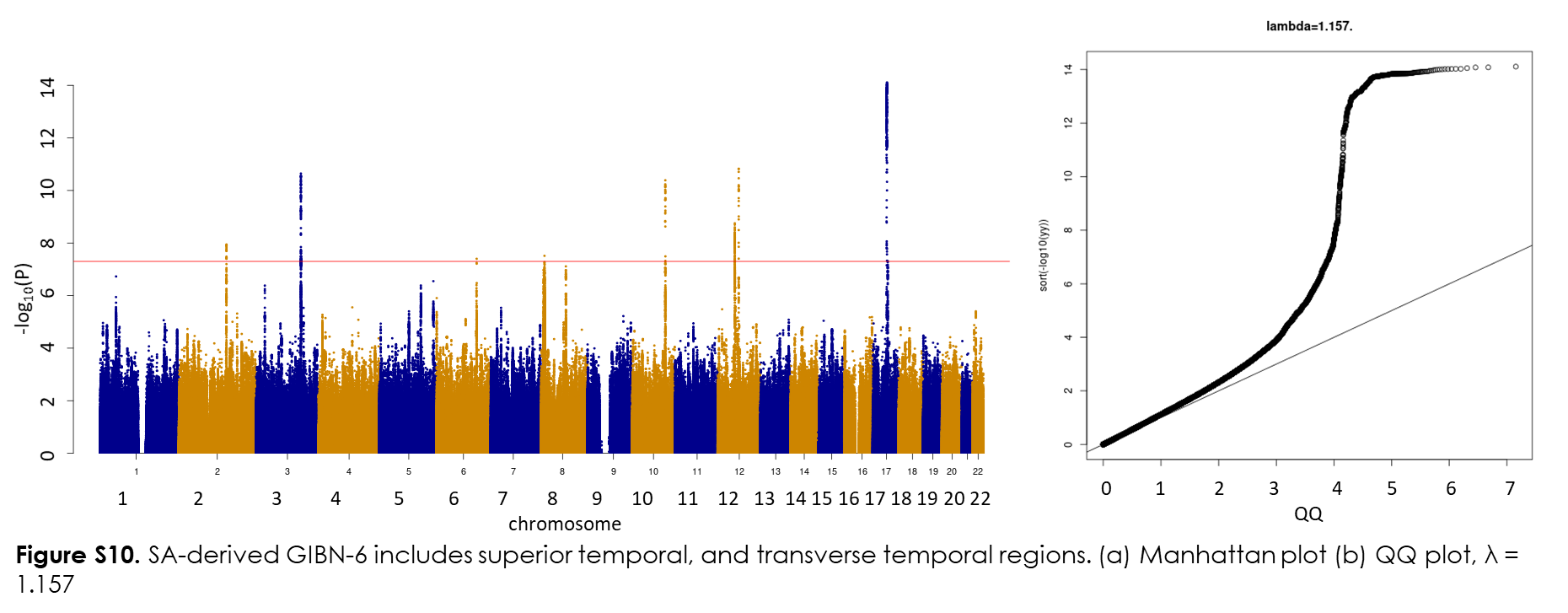
**

**
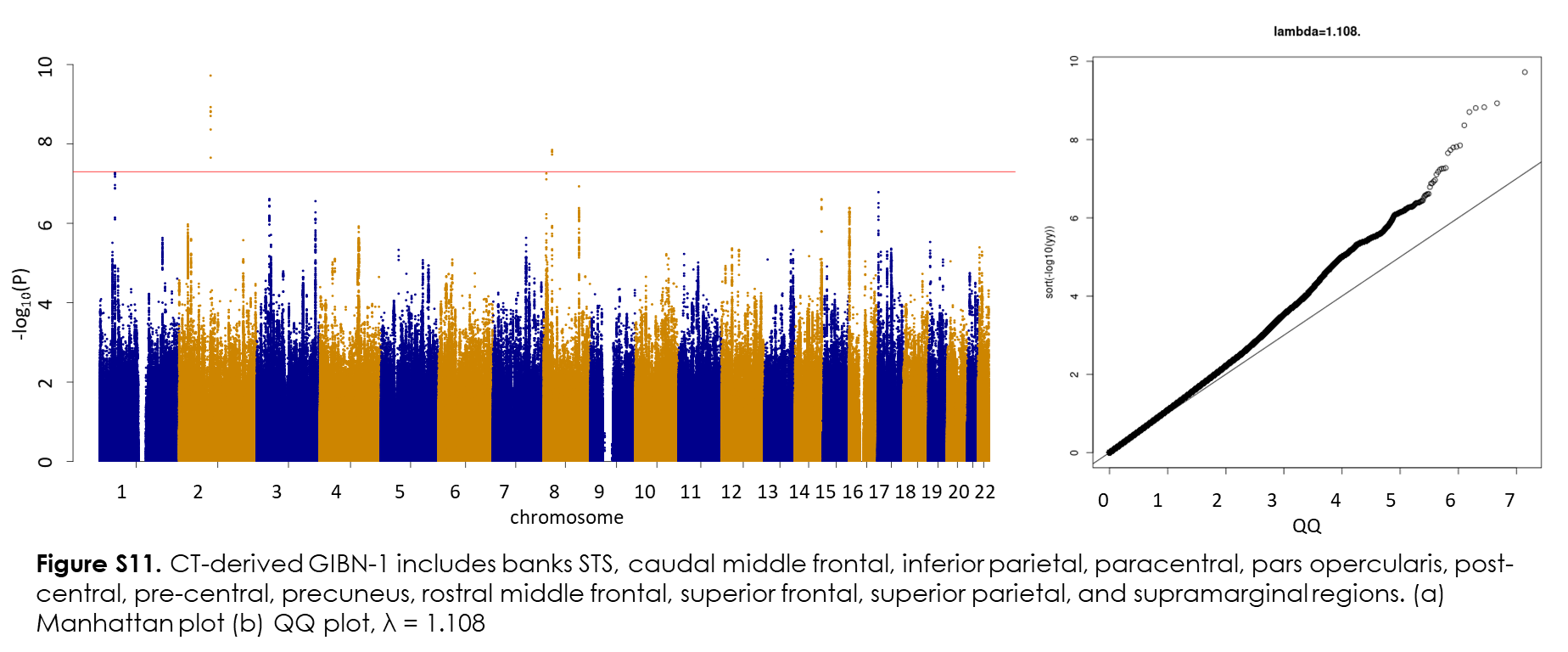
**

**
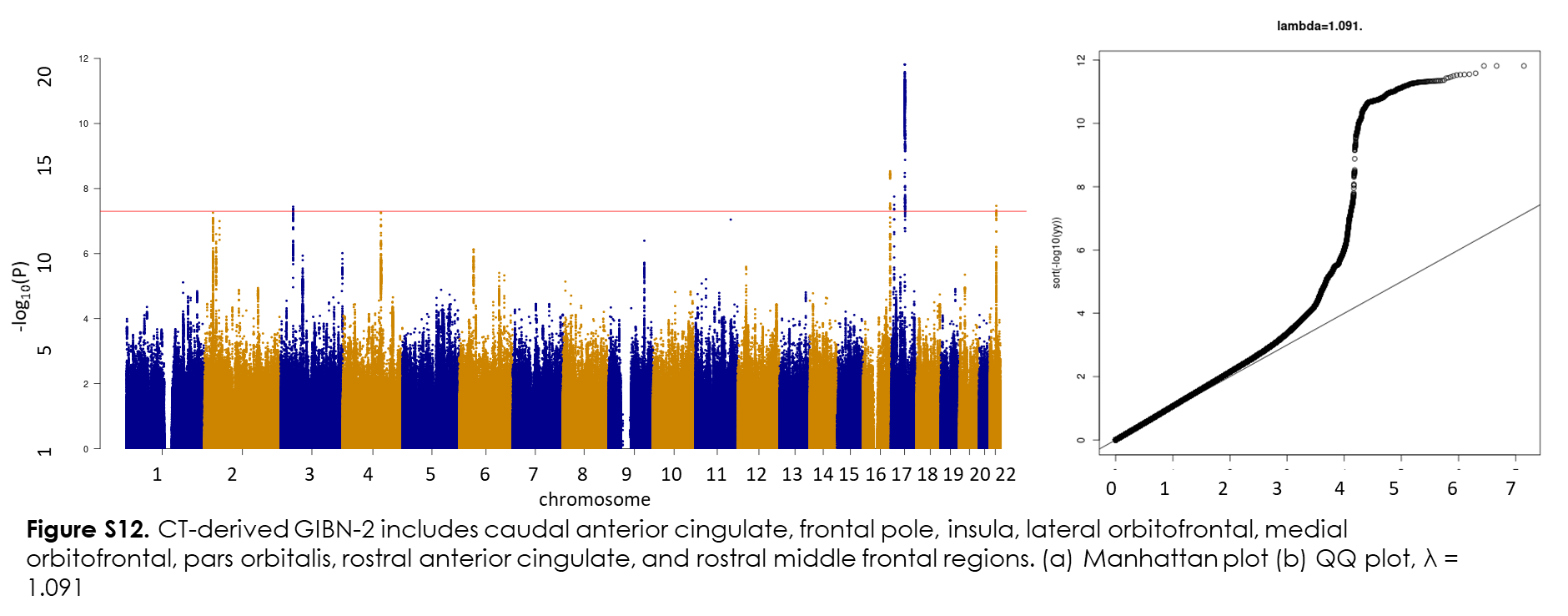
**

**
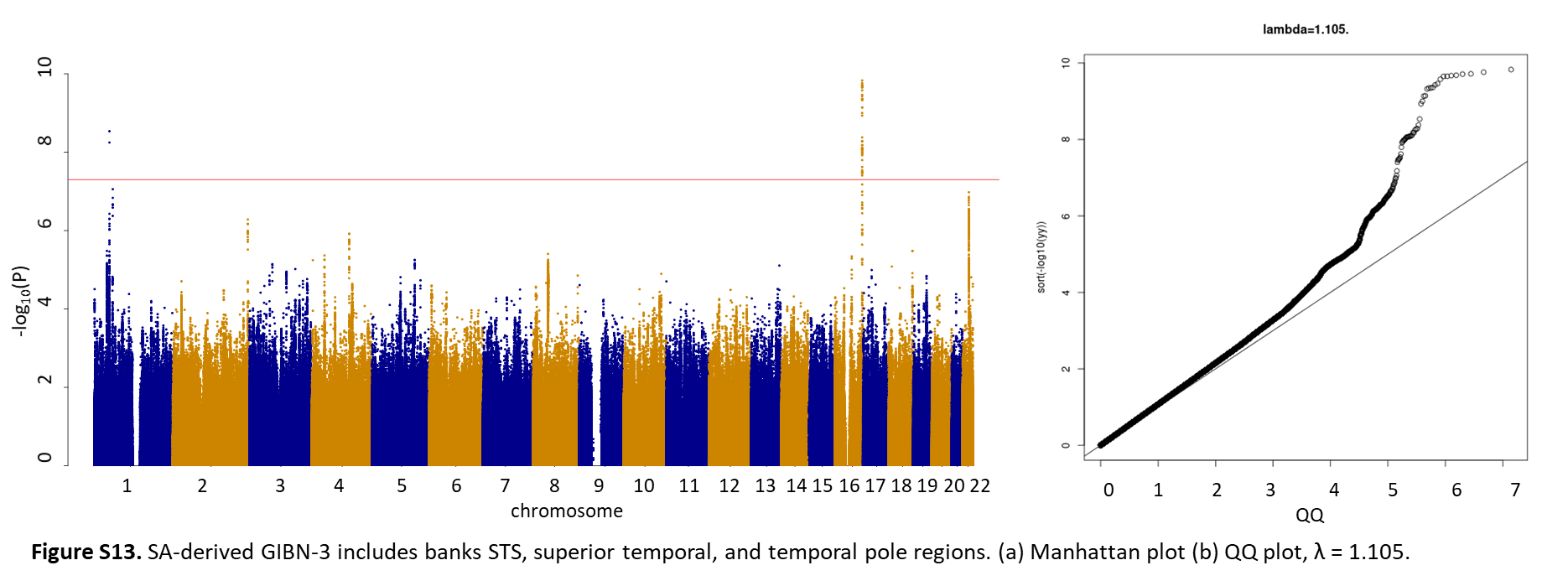
**

**
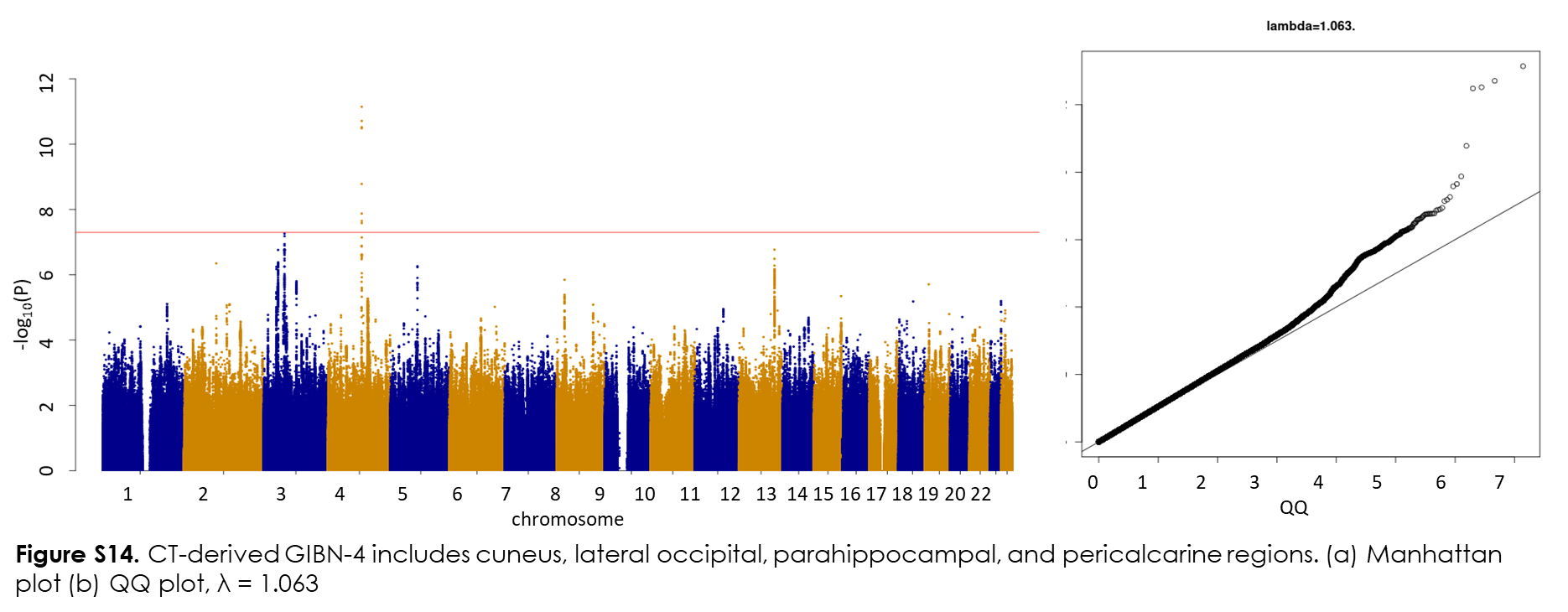
**

**
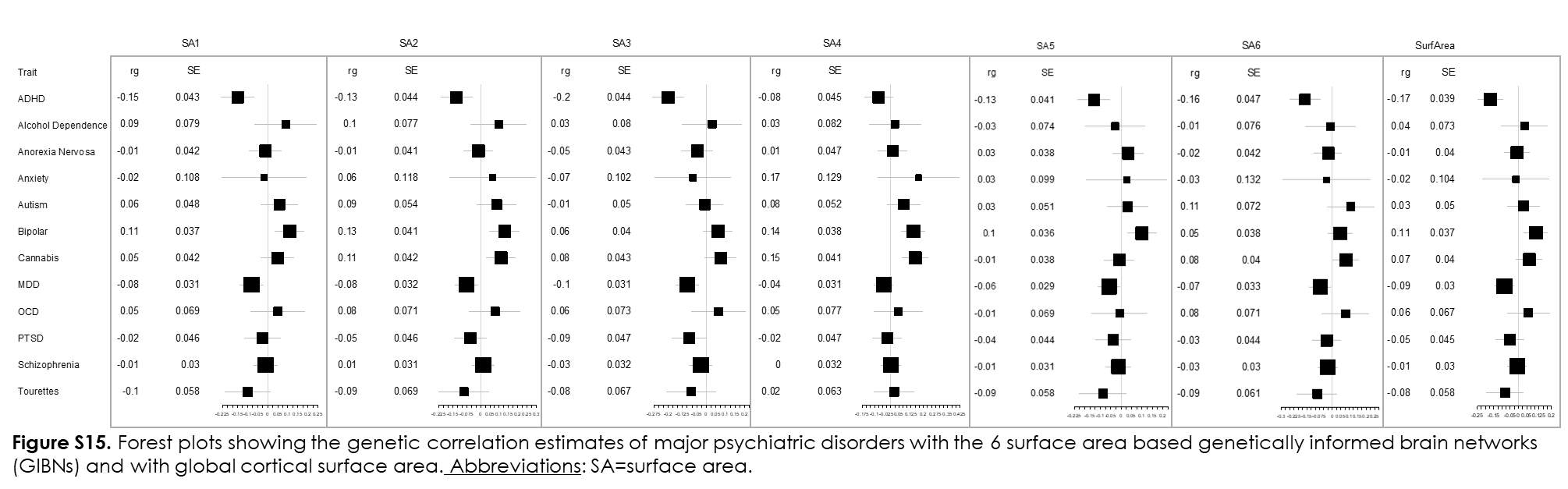
**

**
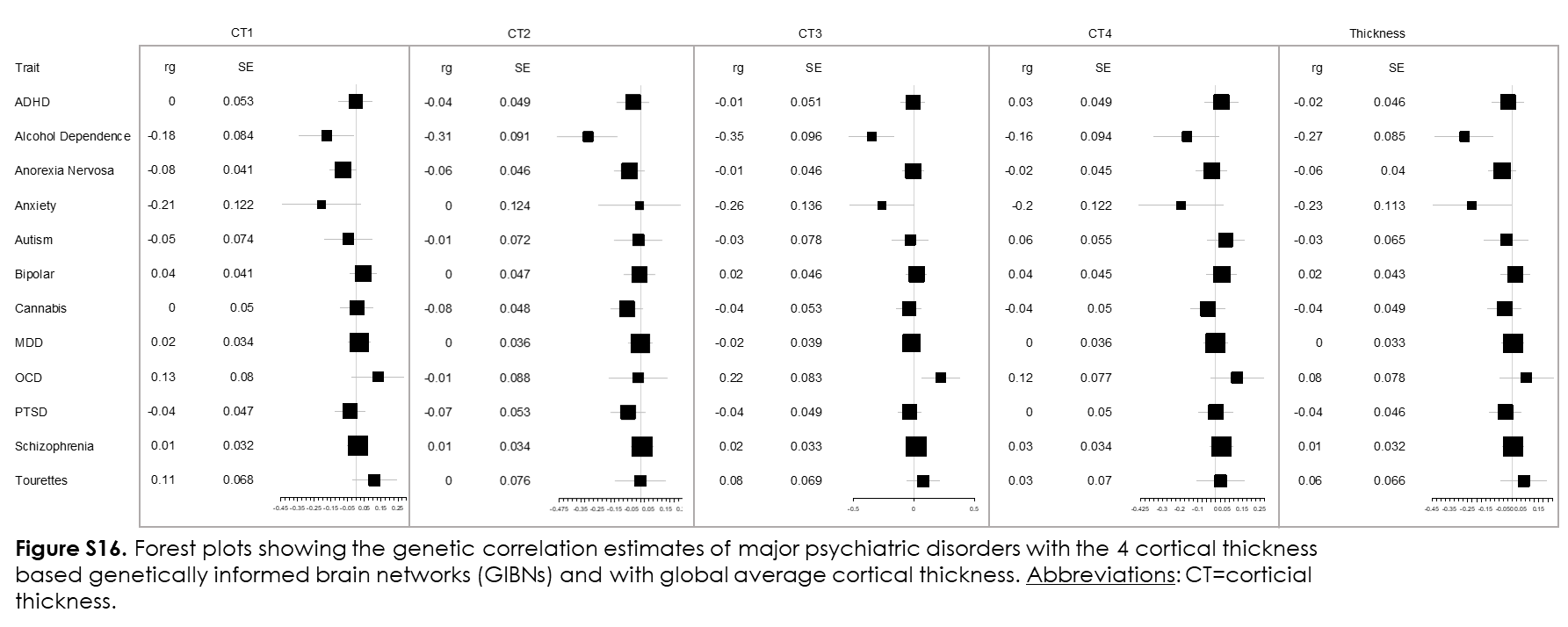
**

**SUPPLEMENTARY TABLES**

**Table S1**. Model Fit Statistics for the genetically informed brain networks (GIBNS) of surface area. The 6 Factor surface area model fit the data the best (lower AIC, lower SRMR, or higher CFI indicate better fit).

| Number of Factors | Cutoff in EFA | AIC | model Ꭓ^2^ | DF | CFI | SRMR |
| --- | --- | --- | --- | --- | --- | --- |
| 6 | **0.5** | **22,712,604.05** | **22,712,478.1** | **237** | **0.92** | **0.062** |
| 5 | 0.5 | 30,128,114.56 | 30,127,992.6 | 264 | 0.91 | 0.064 |
| 3 | 0.5 | 37,107,176.74 | 37,107,062.7 | 321 | 0.89 | 0.064 |
| 6 | 0.3 | 38,521,514.25 | 38,521,322.3 | 465 | 0.91 | 0.056 |
| 9 | 0.3 | 39,497,504.82 | 39,497,276.8 | 481 | 0.91 | 0.055 |
| 4 | 0.3 | 42,768,842.21 | 42,768,674.2 | 511 | 0.90 | 0.058 |
| 7 | 0.3 | 43,175,172.83 | 43,174,968.8 | 493 | 0.90 | 0.060 |
| 5 | 0.3 | 44,590,135.39 | 44,589,955.4 | 471 | 0.90 | 0.059 |
| 3 | 0.3 | 47,348,969.86 | 47,348,817.9 | 519 | 0.89 | 0.061 |
| 2 | 0.5 | 51,228,607.05 | 51,228,473.1 | 494 | 0.88 | 0.071 |
| 1 | 0.5 | 53,156,095.52 | 53,155,963.5 | 495 | 0.87 | 0.073 |
| 2 | 0.3 | 53,561,525.01 | 53,561,385.0 | 525 | 0.88 | 0.071 |
| 1 | 0.3 | 60,204,948.95 | 60,204,812.9 | 527 | 0.86 | 0.077 |

Abbreviations: GIBN=genetically informed brain network; AIC=Akaike Information Criteria; DF = degrees of freedom; CFI=Comparative Fit Index; SRMR=Standardized Root Mean Square Residuals.

**Table S2**. Estimated Loadings for the best fitting 6-GIBN model for surface area (standardized estimates).

| GIBN | Region | Standardized Estimates | p |
| --- | --- | --- | --- |
| SA1 | inferiortemporal | 0.79 | 6.25E-35 |
| SA1 | isthmuscingulate | 0.79 | 5.91E-25 |
| SA1 | postcentral | 0.90 | 8.90E-62 |
| SA1 | precuneus | 0.81 | 4.24E-44 |
| SA1 | superiorparietal | 0.82 | 8.00E-35 |
| SA1 | supramarginal | 0.90 | 5.45E-38 |
| SA1 | temporalpole | 0.70 | 6.73E-21 |
| SA2 | caudalanteriorcingulate | 0.76 | 7.75E-30 |
| SA2 | caudalmiddlefrontal | 0.76 | 4.78E-32 |
| SA2 | medialorbitofrontal | 0.93 | 1.36E-38 |
| SA2 | paracentral | 0.79 | 3.91E-31 |
| SA2 | rostralanteriorcingulate | 0.89 | 5.61E-48 |
| SA3 | bankssts | 0.87 | 4.12E-35 |
| SA3 | inferiorparietal | 0.91 | 1.04E-46 |
| SA3 | middletemporal | 0.96 | 3.97E-41 |
| SA4 | parsopercularis | 0.80 | 5.59E-25 |
| SA4 | parsorbitalis | 0.89 | 9.99E-32 |
| SA4 | parstriangularis | 0.68 | 1.69E-17 |
| SA5 | cuneus | 0.94 | 3.67E-51 |
| SA5 | lateraloccipital | 1.00 | 5.41E-56 |
| SA5 | lingual | 0.89 | 6.58E-39 |
| SA5 | pericalcarine | 0.66 | 1.86E-18 |
| SA6 | superiortemporal | 1.00 | 9.85E-35 |
| SA6 | transversetemporal | 0.83 | 3.96E-30 |

Abbreviations: GIBN=genetically informed brain network; SA=surface area.

**Table S3**. Estimated genetic correlation between GIBNS for the 6-GIBN model for surface area (standardized estimates).

| GIBN1 | GIBN2 | Correlation | p |
| --- | --- | --- | --- |
| SA1 | SA2 | 0.90 | < 5e-300 |
| SA1 | SA3 | 0.90 | 2.51E-213 |
| SA1 | SA4 | 0.84 | 1.16E-113 |
| SA1 | SA5 | 0.76 | 1.21E-94 |
| SA1 | SA6 | 0.78 | 1.70E-74 |
| SA2 | SA3 | 0.76 | 4.11E-79 |
| SA2 | SA4 | 0.91 | 3.77E-151 |
| SA2 | SA5 | 0.72 | 2.78E-74 |
| SA2 | SA6 | 0.80 | 1.67E-122 |
| SA3 | SA4 | 0.69 | 3.58E-39 |
| SA3 | SA5 | 0.61 | 1.29E-33 |
| SA3 | SA6 | 0.66 | 1.81E-27 |
| SA4 | SA5 | 0.73 | 1.32E-51 |
| SA4 | SA6 | 0.85 | 4.71E-49 |
| SA5 | SA6 | 0.64 | 1.62E-47 |

Abbreviations: GIBN=genetically informed brain network; SA=surface area.

**Table S4**. Model Fit Statistics for genetically informed brain networks associated with cortical thickness. The 4-factor cortical thickness model fit the data the best (lower AIC and SRMR, higher CFI indicate better fit).

| Number of Factors | Cutoff in EFA | AIC | model Ꭓ^2^ | DF | CFI | SRMR |
| --- | --- | --- | --- | --- | --- | --- |
| 4 | **0.5** | **17,761,928** | **17,761,812.5** | **267** | **0.93** | **0.077** |
| 5 | 0.5 | 19,714,869 | 19,714,736.7 | 312 | 0.93 | 0.073 |
| 3 | 0.5 | 23,231,378 | 23,231,260.5 | 319 | 0.91 | 0.077 |
| 2 | 0.5 | 24,940,105 | 24,939,991.3 | 349 | 0.91 | 0.081 |
| 1 | 0.5 | 27,175,213 | 27,175,097.1 | 377 | 0.90 | 0.089 |
| 4 | 0.3 | 32,553,940 | 32,553,773.5 | 445 | 0.90 | 0.067 |
| 3 | 0.3 | 35,492,466 | 35,492,316.2 | 453 | 0.89 | 0.078 |
| 5 | 0.3 | 39,453,793 | 39,453,615.0 | 472 | 0.88 | 0.073 |
| 1 | 0.3 | 44,089,256 | 44,089,123.7 | 495 | 0.87 | 0.094 |

Abbreviations: AIC=Akaike Information Criteria; DF=degrees of freedom; CFI=Comparative Fit Index; SRMR=Standardized Root Mean Square Residuals.

**Table S5**. Estimated loadings for the 4-GIBN model for cortical thickness (standardized estimates).

| GIBN | Region | standardized estimates | p |
| --- | --- | --- | --- |
| CT1 | bankssts | 0.83 | 1.74E-19 |
| CT1 | caudalmiddlefrontal | 0.87 | 8.69E-32 |
| CT1 | inferiorparietal | 0.92 | 3.80E-36 |
| CT1 | paracentral | 0.89 | 4.33E-44 |
| CT1 | parsopercularis | 0.90 | 8.81E-27 |
| CT1 | postcentral | 0.75 | 1.14E-15 |
| CT1 | precentral | 0.84 | 2.75E-24 |
| CT1 | precuneus | 0.95 | 1.67E-55 |
| CT1 | rostralmiddlefrontal | 0.54 | 1.37E-08 |
| CT1 | superiorfrontal | 0.93 | 9.93E-39 |
| CT1 | superiorparietal | 0.89 | 4.56E-39 |
| CT1 | supramarginal | 0.92 | 1.38E-30 |
| CT2 | caudalanteriorcingulate | 0.65 | 4.17E-12 |
| CT2 | frontalpole | 0.86 | 1.39E-13 |
| CT2 | insula | 0.81 | 8.09E-20 |
| CT2 | lateralorbitofrontal | 0.87 | 1.52E-20 |
| CT2 | medialorbitofrontal | 0.74 | 1.62E-17 |
| CT2 | parsorbitalis | 0.92 | 2.98E-21 |
| CT2 | rostralanteriorcingulate | 0.67 | 2.42E-10 |
| CT2 | rostralmiddlefrontal | 0.43 | 2.37E-05 |
| CT3 | superiortemporal | 0.98 | 2.10E-32 |
| CT3 | temporalpole | 0.71 | 1.58E-09 |
| CT4 | cuneus | 0.76 | 2.52E-18 |
| CT4 | lateraloccipital | 0.92 | 1.27E-31 |
| CT4 | parahippocampal | 0.40 | 2.55E-06 |
| CT4 | pericalcarine | 0.66 | 2.86E-09 |

Abbreviations: GIBN=genetically informed brain network; ; CT=cortical thickness

**Table S6**. Estimated genetic correlation between GIBNs for the 4-GIBN model for cortical thickness (CT) as standardized estimates (ρ).

| GIBN1 | GIBN2 | ρ | p |
| --- | --- | --- | --- |
| CT1 | CT2 | 0.71 | 2.57E-47 |
| CT1 | CT3 | 0.77 | 4.42E-31 |
| CT1 | CT4 | 0.87 | 2.13E-53 |
| CT2 | CT3 | 0.76 | 8.02E-28 |
| CT2 | CT4 | 0.67 | 1.10E-19 |
| CT3 | CT4 | 0.68 | 3.68E-17 |

Abbreviations: GIBN=genetically informed brain network; CT=cortical thickness

**Table S7**. Model Fit Statistics from the sensitivity analysis of the genetically informed brain networks (GIBNS) of surface area reversing the chromosome set order (exploratory factor analysis in even, confirmatory factor analysis in odd). The 7 Factor surface area model fit the data the best (lower AIC, lower SRMR, or higher CFI indicate better fit).

| Number of Factors | Cutoff in EFA | AIC | model Ꭓ^2^ | DF | CFI | SRMR |
| --- | --- | --- | --- | --- | --- | --- |
| 7 | 0.5 | 35,990,423.2 | 35,990,279.2 | 253 | 0.89 | 0.064 |
| 5 | 0.5 | 42,852,807.3 | 42,852,669.4 | 366 | 0.89 | 0.065 |
| 3 | 0.5 | 44,551,289.0 | 44,551,167.0 | 374 | 0.88 | 0.067 |
| 4 | 0.5 | 46,756,432.4 | 46,756,304.5 | 371 | 0.88 | 0.068 |
| 8 | 0.3 | 53,395,900.2 | 53,395,694.3 | 458 | 0.87 | 0.066 |
| 7 | 0.3 | 53,458,691.3 | 53,458,505.4 | 468 | 0.87 | 0.067 |
| 6 | 0.3 | 54,550,270.2 | 54,550,096.2 | 508 | 0.87 | 0.069 |
| 5 | 0.3 | 55,026,670.5 | 55,026,504.6 | 512 | 0.87 | 0.069 |
| 4 | 0.3 | 56,792,714.5 | 56,792,556,5 | 516 | 0.87 | 0.070 |
| 3 | 0.3 | 57,245,633.6 | 57,245,481,6 | 519 | 0.84 | 0.071 |
| 2 | 0.5 | 65,917,784.2 | 65,917,654.2 | 463 | 0.84 | 0.080 |
| 2 | 0.3 | 67,651,989.8 | 67,651,841.9 | 521 | 0.82 | 0.078 |
| 1 | 0.3 | 75,240,141.9 | 75,240,005.9 | 527 | 0.82 | 0.083 |

Abbreviations: GIBN=genetically informed brain network; AIC=Akaike Information Criteria; DF = degrees of freedom; CFI=Comparative Fit Index; SRMR=Standardized Root Mean Square Residuals.

**Table S8**. Estimated Loadings (standardized estimates) for the best fitting 7-GIBN model for surface area from the sensitivity analyses reversing the chromosome set order (exploratory factor analysis in even, confirmatory factor analysis in odd).

| GIBN | Region | Standardized Estimates | p |
| --- | --- | --- | --- |
| SA1 | bankssts | 0.74 | 3.51E-21 |
| SA1 | fusiform | 0.86 | 2.86E-45 |
| SA1 | inferiorparietal | 0.92 | 1.22E-54 |
| SA1 | inferiortemporal | 0.89 | 1.04E-56 |
| SA1 | middletemporal | 0.90 | 2.46E-50 |
| SA1 | parahippocampal | 0.77 | 3.18E-28 |
| SA1 | supramarginal | 0.64 | 9.31E-05 |
| SA2 | cuneus | 1.00 | 2.41E-41 |
| SA2 | lateraloccipital | 0.99 | 3.24E-44 |
| SA2 | lingual | 0.79 | 1.69E-28 |
| SA2 | pericalcarine | 0.66 | 2.74E-23 |
| SA3 | insula | 0.82 | 8.57E-28 |
| SA3 | parsopercularis | 0.76 | 5.60E-19 |
| SA3 | parstriangularis | 0.69 | 1.62E-20 |
| SA3 | superiortemporal | 0.89 | 3.74E-34 |
| SA3 | transversetemporal | 0.71 | 1.81E-23 |
| SA4 | medialorbitofrontal | 0.95 | 2.59E-46 |
| SA4 | paracentral | 0.80 | 3.36E-24 |
| SA4 | rostralanteriorcingulate | 0.89 | 1.85E-42 |
| SA5 | lateralorbitofrontal | 0.93 | 2.97E-47 |
| SA5 | parsorbitalis | 0.88 | 2.75E-36 |
| SA6 | postcentral | 0.95 | 4.36E-46 |
| SA6 | precentral | 0.88 | 8.38E-38 |
| SA6 | rostralanteriorcingulate | 0.20 | 2.23E-01 |
| SA7 | caudalanteriorcingulate | 0.83 | 6.55E-21 |
| SA7 | posteriorcingulate | 0.89 | 4.31E-24 |

Abbreviations: GIBN=genetically informed brain network; SA=surface area.

**Table S9**. Estimated genetic correlation (standardized estimates) between GIBNS for the 7-GIBN model for surface area from the sensitivity analyses reversing the chromosome set order (exploratory factor analysis in even, confirmatory factor analysis in odd).

| GIBN1 | GIBN2 | Correlation | p |
| --- | --- | --- | --- |
| SA1 | SA2 | 0.55 | 5.12E-30 |
| SA1 | SA3 | 0.82 | 9.73E-101 |
| SA1 | SA4 | 0.80 | 4.26E-121 |
| SA1 | SA5 | 0.75 | 2.66E-85 |
| SA1 | SA6 | 0.86 | 3.64E-139 |
| SA1 | SA7 | 0.85 | 5.14E-98 |
| SA2 | SA3 | 0.53 | 3.95E-22 |
| SA2 | SA4 | 0.58 | 2.79E-35 |
| SA2 | SA5 | 0.56 | 1.66E-23 |
| SA2 | SA6 | 0.70 | 2.57E-59 |
| SA2 | SA7 | 0.56 | 2.29E-21 |
| SA3 | SA4 | 0.85 | 5.51E-111 |
| SA3 | SA5 | 0.87 | 9.73E-153 |
| SA3 | SA6 | 0.82 | 1.75E-97 |
| SA3 | SA7 | 0.78 | 1.64E-52 |
| SA4 | SA5 | 0.79 | 3.56E-88 |
| SA4 | SA6 | 0.85 | 9.10E-119 |
| SA4 | SA7 | 0.88 | 2.71E-129 |
| SA5 | SA6 | 0.76 | 7.52E-73 |
| SA5 | SA7 | 0.73 | 9.66E-41 |
| SA6 | SA7 | 0.77 | 3.21E-49 |

Abbreviations: GIBN=genetically informed brain network; SA=surface area.

**Table S10**. Model Fit Statistics for the sensitivity analyses of genetically informed brain networks associated with cortical thickness reversing the chromosome set order (exploratory factor analysis in even, confirmatory factor analysis in odd). The 4 Factor surface area model fit the data the best (lower AIC, lower SRMR, or higher CFI indicate better fit).

| Number of Factors | Cutoff in EFA | AIC | model Ꭓ^2^ | DF | CFI | SRMR |
| --- | --- | --- | --- | --- | --- | --- |
| 4 | **0.3** | **58,699,212.8** | **58,699,046.8** | **512** | **0.86** | **0.077** |
| 2 | 0.3 | 63,055,094.7 | 63,054,940.7 | 518 | 0.85 | 0.082 |
| 3 | 0.3 | 64,190,549.8 | 64,190,383.8 | 512 | 0.85 | 0.080 |
| 1 | 0.5 | 65,300,561.0 | 65,300,433.0 | 464 | 0.83 | 0.105 |
| 2 | 0.5 | 69,957,082.7 | 69,956,952.7 | 463 | 0.83 | 0.092 |
| 1 | 0.3 | 78,215,831.1 | 78,215,695.1 | 527 | 0.82 | 0.104 |

Abbreviations: AIC=Akaike Information Criteria; DF=degrees of freedom; CFI=Comparative Fit Index; SRMR=Standardized Root Mean Square Residuals.

**Table S11**. Estimated loadings (standardized estimates) for the 4-GIBN model for cortical thickness from the sensitivityanalyses reversing the chromosome set order (exploratory factor analysis in even, confirmatory factor analysis in odd.

| GIBN | Region | standardized estimates | P |
| --- | --- | --- | --- |
| CT1 | bankssts | 0.87 | 2.352E-28 |
| CT1 | caudalmiddlefrontal | 0.84 | 1.58E-22 |
| CT1 | cuneus | 0.62 | 1.48E-09 |
| CT1 | fusiform | 0.92 | 1.77E-41 |
| CT1 | lateraloccipital | 0.81 | 9.33E-37 |
| CT1 | lingual | 0.74 | 9.34E-24 |
| CT1 | middletemporal | -0.09 | 7.14E-01 |
| CT1 | paracentral | 0.76 | 2.76E-26 |
| CT1 | parsopercularis | 0.60 | 1.37E-08 |
| CT1 | parstriangularis | 0.44 | 4.45E-07 |
| CT1 | pericalcarine | 0.57 | 8.38E-10 |
| CT1 | postcentral | 0.75 | 1.78E-25 |
| CT1 | precentral | 0.84 | 1.55E-30 |
| CT1 | precuneus | 0.88 | 1.22E-42 |
| CT1 | rostralmiddlefrontal | 0.36 | 3.44E-05 |
| CT1 | superiorfrontal | 0.61 | 6.62E-14 |
| CT1 | superiorparietal | 0.88 | 2.83E-47 |
| CT1 | superiortemporal | 0.77 | 5.02E-32 |
| CT1 | supramarginal | 0.94 | 2.18E-51 |
| CT1 | transversetemporal | 0.74 | 3.65E-24 |
| CT2 | caudalanteriorcingulate | 0.53 | 5.72E-09 |
| CT2 | frontalpole | 0.80 | 4.09E-10 |
| CT2 | fusiform | -0.06 | 7.51E-01 |
| CT2 | insula | 0.77 | 1.53E-17 |
| CT2 | isthmuscingulate | 0.56 | 5.86E-17 |
| CT2 | lateralorbitofrontal | 1.03 | 6.07E-07 |
| CT2 | medialorbitofrontal | 0.73 | 4.20E-12 |
| CT2 | parsopercularis | 0.34 | 9.70E-04 |
| CT2 | parsorbitalis | 0.57 | 6.03E-04 |
| CT2 | parstriangularis | 0.51 | 5.13E-07 |
| CT2 | posteriorcingulate | 0.75 | 7.43E-24 |
| CT2 | precuneus | 0.65 | 6.07E-08 |
| CT2 | rostralanteriorcingulate | 0.61 | 2.10E-09 |
| CT2 | rostralmiddlefrontal | 0.34 | 2.86E-04 |
| CT3 | fusiform | 0.94 | 6.23E-06 |
| CT3 | inferiortemporal | 0.78 | 1.06E-16 |
| CT3 | lateralorbitofrontal | -0.21 | 2.69E-01 |
| CT3 | middletemporal | 0.97 | 1.12E-04 |
| CT3 | parsorbitalis | 0.29 | 7.17E-02 |
| CT3 | supramarginal | 0.54 | 8.28E-06 |
| CT4 | cuneus | 0.23 | 9.60E-02 |
| CT4 | entorhinal | 0.72 | 6.02E-06 |
| CT4 | parahippocampal | 0.47 | 1.26E-04 |

Abbreviations: GIBN=genetically informed brain network; ; CT=cortical thickness

**Table S12**. Estimated genetic correlation (standardized estimates) between GIBNS for the 4-GIBN model for cortical Thickness from the sensitivity analyses reversing the chromosome set order (exploratory factor analysis in even, confirmatory factor analysis in odd.

| GIBN1 | GIBN2 | Correlation | p |
| --- | --- | --- | --- |
| CT1 | CT2 | 0.69 | 9.26E-40 |
| CT1 | CT3 | 0.85 | 1.27E-58 |
| CT1 | CT4 | 0.47 | 2.39E-04 |
| CT2 | CT3 | 0.80 | 1.04E-27 |
| CT2 | CT4 | 0.51 | 1.19E-05 |
| CT3 | CT4 | 0.66 | 4.77E-07 |

Abbreviations: GIBN=genetically informed brain network; CT=cortical thickness

**Table S13**. Independent GWS loci associated with GIBNs as identified by FUMA.

| **GIBN** | **rsID** | **Chr** | **Pos** | **Beta** | **p** |
| --- | --- | --- | --- | --- | --- |
| **SA2** | rs4273712 | 6 | 126964510 | -0.29 | 1.66E-21 |
| **SA1** | rs62057153 | 17 | 43904528 | 0.28 | 8.45E-21 |
| **SA5** | rs55638417 | 17 | 43900434 | 0.28 | 8.60E-21 |
| **SA5** | rs74580701 | 6 | 127000881 | 0.66 | 6.64E-18 |
| **SA2** | rs79600142 | 17 | 43897722 | 0.26 | 1.13E-17 |
| **SA3** | rs56319902 | 17 | 43871982 | 0.26 | 5.22E-17 |
| **SA5** | rs73313052 | 14 | 59625997 | 0.28 | 7.48E-17 |
| **SA2** | rs7312464 | 12 | 66374247 | 0.20 | 7.78E-16 |
| **SA6** | rs9896243 | 17 | 44826056 | 0.23 | 7.75E-15 |
| **SA4** | rs55663797 | 17 | 43544379 | 0.27 | 2.27E-13 |
| **SA1** | rs11759026 | 6 | 126792095 | -0.19 | 3.48E-13 |
| **SA1** | rs34464850 | 3 | 141721762 | -0.21 | 3.60E-13 |
| **SA5** | rs6788676 | 3 | 104674040 | -0.17 | 3.89E-13 |
| **SA3** | rs8756 | 12 | 66359752 | 0.18 | 5.84E-13 |
| **SA1** | rs7312464 | 12 | 66374247 | 0.16 | 6.47E-13 |
| **CT2** | rs2316766 | 17 | 43919068 | -0.28 | 1.54E-12 |
| **SA4** | rs2279829 | 3 | 147106319 | -0.24 | 4.21E-12 |
| **CT4** | rs13107325 | 4 | 103188709 | 0.44 | 7.19E-12 |
| **SA3** | rs34464850 | 3 | 141721762 | -0.21 | 9.61E-12 |
| **SA1** | rs1628768 | 10 | 105012994 | -0.17 | 1.07E-11 |
| **SA6** | rs10878349 | 12 | 66327632 | 0.16 | 1.49E-11 |
| **SA6** | rs34464850 | 3 | 141721762 | -0.20 | 2.27E-11 |
| **SA6** | rs1163249 | 10 | 104909890 | 0.18 | 4.10E-11 |
| **CT3** | rs12711473 | 16 | 87224293 | 0.19 | 1.48E-10 |
| **CT1** | rs11692435 | 2 | 98275354 | 0.30 | 1.90E-10 |
| **SA4** | rs11759026 | 6 | 126792095 | -0.19 | 2.84E-10 |
| **SA4** | rs4924345 | 15 | 39639898 | 0.32 | 3.78E-10 |
| **SA3** | rs4917384 | 10 | 104995788 | 0.15 | 4.13E-10 |
| **SA3** | rs3847535 | 11 | 92558024 | 0.15 | 5.62E-10 |
| **SA2** | rs1628768 | 10 | 105012994 | -0.16 | 7.21E-10 |
| **SA1** | rs7082934 | 10 | 21881379 | 0.13 | 8.26E-10 |
| **SA5** | rs3858368 | 11 | 92553286 | 0.14 | 1.35E-09 |
| **SA2** | rs7778997 | 7 | 156183533 | -0.36 | 1.38E-09 |
| **SA5** | rs13135092 | 4 | 103198082 | -0.23 | 1.41E-09 |
| **SA2** | rs7297175 | 12 | 56473808 | 0.14 | 1.43E-09 |
| **SA5** | rs3754356 | 1 | 228270964 | 0.16 | 1.61E-09 |
| **SA4** | rs11012732 | 10 | 21830104 | 0.17 | 1.63E-09 |
| **SA6** | rs11170566 | 12 | 53907067 | -0.19 | 1.78E-09 |
| **SA2** | rs11079849 | 17 | 47090785 | -0.15 | 1.91E-09 |
| **SA4** | rs2271386 | 3 | 141712708 | -0.20 | 2.12E-09 |
| **SA2** | rs3817176 | 3 | 141712780 | -0.17 | 2.64E-09 |
| **CT3** | rs61784835 | 1 | 47974123 | 0.17 | 2.92E-09 |
| **CT2** | rs12711473 | 16 | 87224293 | 0.17 | 2.94E-09 |
| **SA2** | rs4792721 | 17 | 16006827 | 0.13 | 3.60E-09 |
| **SA1** | rs3006933 | 1 | 243659727 | -0.13 | 4.08E-09 |
| **SA2** | rs2490272 | 6 | 108895386 | -0.13 | 4.41E-09 |
| **SA4** | rs7711765 | 5 | 170832637 | -0.16 | 4.52E-09 |
| **SA4** | rs113343136 | 9 | 98314306 | -0.26 | 4.88E-09 |
| **SA1** | rs7563432 | 2 | 48281138 | 0.13 | 6.18E-09 |
| **SA5** | rs10765918 | 11 | 12071855 | 0.15 | 6.24E-09 |
| **SA4** | rs7312464 | 12 | 66374247 | 0.15 | 7.94E-09 |
| **SA3** | rs78200999 | 7 | 155996837 | -0.14 | 9.78E-09 |
| **SA5** | rs10878349 | 12 | 66327632 | 0.12 | 1.06E-08 |
| **SA6** | rs13035861 | 2 | 150040224 | -0.12 | 1.13E-08 |
| **SA3** | rs2301718 | 4 | 106009763 | -0.16 | 1.39E-08 |
| **CT1** | rs3200031 | 8 | 26227484 | -0.24 | 1.41E-08 |
| **CT2** | rs12602519 | 17 | 10027480 | -0.16 | 1.77E-08 |
| **SA5** | rs1628768 | 10 | 105012994 | -0.13 | 1.77E-08 |
| **SA3** | rs28457693 | 9 | 98217348 | -0.20 | 2.18E-08 |
| **SA1** | rs7975351 | 12 | 53902190 | -0.12 | 2.93E-08 |
| **SA1** | rs12630663 | 3 | 28007315 | -0.12 | 3.02E-08 |
| **SA6** | rs9969436 | 8 | 10842659 | -0.12 | 3.07E-08 |
| **SA5** | rs6701689 | 1 | 2020343 | -0.13 | 3.19E-08 |
| **CT2** | rs1004763 | 22 | 38474952 | -0.15 | 3.41E-08 |
| **SA5** | rs56007616 | 3 | 118994959 | 0.18 | 3.61E-08 |
| **CT2** | rs533577 | 3 | 39489651 | 0.15 | 3.61E-08 |
| **SA4** | rs7297175 | 12 | 56473808 | 0.14 | 3.83E-08 |
| **SA6** | rs4273712 | 6 | 126964510 | -0.13 | 3.98E-08 |
| **SA5** | rs7570830 | 2 | 61745528 | -0.11 | 4.01E-08 |
| **SA3** | rs2299148 | 7 | 42023400 | -0.14 | 4.43E-08 |
| **SA5** | rs2802292 | 6 | 108908518 | -0.11 | 4.63E-08 |
| **SA1** | rs10109434 | 8 | 41169496 | 0.12 | 4.72E-08 |
| **SA5** | rs12357321 | 10 | 21790476 | 0.12 | 4.74E-08 |
| **SA6** | rs9909861 | 17 | 47079416 | -0.12 | 4.89E-08 |

**Table S14 Correlation between CT and SA GIBNS**

| **GIBN1** | **GIBN2** | **ρ** | **p** |
| --- | --- | --- | --- |
| CT1 | SA1 | -0.2842 | 3.88E-09 |
| CT1 | SA2 | -0.2031 | 0.0002 |
| CT1 | SA3 | -0.1472 | 0.0042 |
| CT1 | SA4 | -0.1872 | 0.0001 |
| CT1 | SA5 | -0.2263 | 3.82E-07 |
| CT1 | SA6 | -0.2233 | 0.0003 |
| CT2 | SA1 | -0.3923 | 3.02E-17 |
| CT2 | SA2 | -0.4289 | 1.77E-19 |
| CT2 | SA3 | -0.2958 | 3.11E-08 |
| CT2 | SA4 | -0.3541 | 1.73E-12 |
| CT2 | SA5 | -0.3128 | 5.84E-13 |
| CT2 | SA6 | -0.3048 | 8.81E-09 |
| CT3 | SA1 | -0.1999 | 8.70E-05 |
| CT3 | SA2 | -0.1633 | 0.0027 |
| CT3 | SA3 | -0.095 | 0.0736 |
| CT3 | SA4 | -0.0904 | 0.0992 |
| CT3 | SA5 | -0.1475 | 0.0039 |
| CT3 | SA6 | -0.1758 | 0.0076 |
| CT4 | SA1 | -0.2311 | 5.29E-06 |
| CT4 | SA2 | -0.1957 | 0.0002 |
| CT4 | SA3 | -0.1653 | 0.0016 |
| CT4 | SA4 | -0.1618 | 0.0011 |
| CT4 | SA5 | -0.0763 | 0.1318 |
| CT4 | SA6 | -0.2117 | 3.14E-05 |

Abbreviations: GIBN=genetically informed brain network; CT=cortical thickness derived GIBNS; SA=surface area derived GIBNS;

**Table S15**. LDSC estimates of the pairwise genetic correlation between SA-derived GIBNs and psychiatric disorders. Nominally significant associations are indicated in bold. Genetic correlation between global SA and psychiatric disorders included for comparison.

| **p1** | **p2** | **rg** | **p** | **FDR** | **p1** | **p2** | **rg** | **p** | **FDR** |
| --- | --- | --- | --- | --- | --- | --- | --- | --- | --- |
| SA1 | **ADHD** | **-0.15** | **6.00E-04** | **0.014** | **SA5** | **ADHD** | **-0.13** | **0.0013** | **0.017** |
|  | Alcohol Dependence | 0.090 | 0.25 | 0.58 |  | Alcohol Dependence | -0.029 | 0.69 | 0.91 |
|  | Anorexia Nervosa | -0.011 | 0.79 | 0.95 |  | Anorexia Nervosa | 0.034 | 0.38 | 0.71 |
|  | Anxiety | -0.017 | 0.88 | 0.96 |  | Anxiety | 0.028 | 0.78 | 0.95 |
|  | Autism | 0.058 | 0.23 | 0.57 |  | Autism | 0.031 | 0.55 | 0.85 |
|  | **Bipolar** | **0.11** | **0.0047** | **0.043** |  | **Bipolar** | **0.10** | **0.0039** | **0.039** |
|  | Cannabis | 0.049 | 0.24 | 0.57 |  | Cannabis | -0.0089 | 0.81 | 0.95 |
|  | **MDD** | **-0.080** | **0.0098** | **0.078** |  | MDD | -0.057 | 0.046 | 0.26 |
|  | OCD | 0.051 | 0.46 | 0.77 |  | OCD | -0.0073 | 0.92 | 0.99 |
|  | PTSD | -0.025 | 0.59 | 0.88 |  | PTSD | -0.038 | 0.39 | 0.71 |
|  | Schizophrenia | -0.011 | 0.73 | 0.93 |  | Schizophrenia | -0.013 | 0.67 | 0.91 |
|  | Tourette’s | -0.099 | 0.087 | 0.37 |  | Tourette’s | -0.086 | 0.142 | 0.44 |
| SA2 | **ADHD** | **-0.13** | **0.0038** | **0.039** | **SA6** | **ADHD** | **-0.16** | **8.00E-04** | **0.014** |
|  | Alcohol Dependence | 0.097 | 0.21 | 0.54 |  | Alcohol Dependence | -0.010 | 0.89 | 0.97 |
|  | Anorexia Nervosa | -0.012 | 0.78 | 0.95 |  | Anorexia Nervosa | -0.023 | 0.59 | 0.88 |
|  | Anxiety | 0.065 | 0.58 | 0.88 |  | Anxiety | -0.034 | 0.80 | 0.95 |
|  | Autism | 0.086 | 0.114 | 0.40 |  | Autism | 0.11 | 0.124 | 0.41 |
|  | **Bipolar** | **0.13** | **0.0018** | **0.022** |  | Bipolar | 0.047 | 0.21 | 0.54 |
|  | Cannabis | 0.11 | 0.011 | 0.080 |  | Cannabis | 0.082 | 0.039 | 0.23 |
|  | MDD | -0.075 | 0.020 | 0.14 |  | MDD | -0.071 | 0.030 | 0.20 |
|  | OCD | 0.078 | 0.27 | 0.58 |  | OCD | 0.079 | 0.27 | 0.58 |
|  | PTSD | -0.054 | 0.23 | 0.57 |  | PTSD | -0.033 | 0.45 | 0.77 |
|  | Schizophrenia | 0.014 | 0.66 | 0.91 |  | Schizophrenia | -0.026 | 0.39 | 0.71 |
|  | Tourette’s | -0.090 | 0.19 | 0.51 |  | Tourette’s | -0.088 | 0.153 | 0.45 |
| SA3 | **ADHD** | **-0.20** | **3.29E-06** | **0.00040** | **Global Surface Area** | **ADHD** | **-0.1702** | **1.30E-05** |  |
|  | Alcohol Dependence | 0.031 | 0.70 | 0.91 |  | Alcohol Dependence | 0.0394 | 0.5875 |  |
|  | Anorexia Nervosa | -0.047 | 0.27 | 0.58 |  | Anorexia Nervosa | -0.0079 | 0.8421 |  |
|  | Anxiety | -0.070 | 0.50 | 0.82 |  | Anxiety | -0.0151 | 0.8847 |  |
|  | Autism | -0.0084 | 0.87 | 0.96 |  | Autism | 0.0313 | 0.5289 |  |
|  | Bipolar | 0.061 | 0.131 | 0.41 |  | **Bipolar** | **0.1061** | **0.0037** |  |
|  | Cannabis | 0.075 | 0.085 | 0.37 |  | Cannabis | 0.066 | 0.1008 |  |
|  | **MDD** | **-0.10** | **0.0011** | **0.017** |  | **MDD** | **-0.0854** | **0.0044** |  |
|  | OCD | 0.064 | 0.38 | 0.71 |  | OCD | 0.0604 | 0.3642 |  |
|  | PTSD | -0.089 | 0.056 | 0.29 |  | PTSD | -0.0518 | 0.2449 |  |
|  | Schizophrenia | -0.029 | 0.35 | 0.71 |  | Schizophrenia | -0.008 | 0.7923 |  |
|  | Tourette’s | -0.079 | 0.24 | 0.57 |  | Tourette’s | -0.0807 | 0.1605 |  |
| SA4 | ADHD | -0.076 | 0.093 | 0.39 |  | | | | |
|  | Alcohol Dependence | 0.026 | 0.75 | 0.94 |  |  |  |  |  |
|  | Anorexia Nervosa | 0.012 | 0.81 | 0.95 |  |  |  |  |  |
|  | Anxiety | 0.17 | 0.18 | 0.49 |  |  |  |  |  |
|  | Autism | 0.080 | 0.125 | 0.41 |  |  |  |  |  |
|  | **Bipolar** | **0.14** | **3.00E-04** | **0.012** |  |  |  |  |  |
|  | **Cannabis** | **0.15** | **4.00E-04** | **0.012** |  |  |  |  |  |
|  | MDD | -0.044 | 0.152 | 0.45 |  |  |  |  |  |
|  | OCD | 0.046 | 0.55 | 0.85 |  |  |  |  |  |
|  | PTSD | -0.022 | 0.64 | 0.91 |  |  |  |  |  |
|  | Schizophrenia | 0.0015 | 0.96 | 0.99 |  |  |  |  |  |
|  | Tourette’s | 0.021 | 0.74 | 0.94 |  |  |  |  |  |

Abbreviations: LDSC=LD score regression; GIBN=genetically informed brain network; SA- =surface area derived GIBNS; r_g_=genetic correlation; ADHD=Attention Deficit Hyperactivity Disorder; MDD=Major Depressive Disorder; P_FDR_ False Discovery Rate corrected significance value.

**Table S16**. LDSC estimates of the pairwise genetic correlation between CT-derived GIBNs and psychiatric disorders. Nominally significant associations are indicated in bold. Genetic correlation between global mean CT and psychiatric disorders included for comparison.

| **p1** | **p2** | **rg** | **p** | **FDR** | **p1** | **p2** | **rg** | **p** | **FDR** |
| --- | --- | --- | --- | --- | --- | --- | --- | --- | --- |
| CT1 | ADHD | -0.0032 | 0.95 | 0.99 | CT4 | ADHD | 0.032 | 0.51 | 0.83 |
|  | **Alcohol Dependence** | **-0.177** | **0.035** | **0.22** |  | Alcohol Dependence | -0.163 | 0.084 | 0.37 |
|  | Anorexia Nervosa | -0.078 | 0.060 | 0.30 |  | Anorexia Nervosa | -0.022 | 0.63 | 0.90 |
|  | Anxiety | -0.208 | 0.087 | 0.37 |  | Anxiety | -0.196 | 0.11 | 0.40 |
|  | Autism | -0.047 | 0.53 | 0.84 |  | Autism | 0.056 | 0.31 | 0.64 |
|  | Bipolar | 0.042 | 0.31 | 0.64 |  | Bipolar | 0.035 | 0.44 | 0.77 |
|  | Cannabis | 0.004 | 0.94 | 0.99 |  | Cannabis | -0.044 | 0.37 | 0.71 |
|  | MDD | 0.018 | 0.61 | 0.89 |  | MDD | -0.00060 | 0.99 | 0.99 |
|  | OCD | 0.13 | 0.11 | 0.40 |  | OCD | 0.12 | 0.13 | 0.41 |
|  | PTSD | -0.037 | 0.43 | 0.77 |  | PTSD | 0.0012 | 0.98 | 0.99 |
|  | Schizophrenia | 0.012 | 0.71 | 0.91 |  | Schizophrenia | 0.033 | 0.34 | 0.69 |
|  | Tourette’s | 0.11 | 0.11 | 0.40 |  | Tourette’s | 0.027 | 0.70 | 0.91 |
| CT2 | ADHD | -0.043 | 0.38 | 0.71 | Average Thickness | ADHD | -0.0235 | 0.6125 |  |
|  | **Alcohol Dependence** | **-0.307** | **7.00E-04** | **0.014** |  | **Alcohol Dependence** | **-0.2684** | **0.0015** |  |
|  | Anorexia Nervosa | -0.063 | 0.17 | 0.49 |  | Anorexia | -0.0562 | 0.1639 |  |
|  | Anxiety | -0.0050 | 0.97 | 0.99 |  | anxiety | -0.2271 | 0.0451 |  |
|  | Autism | -0.013 | 0.86 | 0.96 |  | Autism | -0.0332 | 0.6095 |  |
|  | Bipolar | -0.0044 | 0.93 | 0.99 |  | Bipolar | 0.017 | 0.6938 |  |
|  | Cannabis | -0.079 | 0.10 | 0.40 |  | Cannabis | -0.0424 | 0.3831 |  |
|  | MDD | 0.00030 | 0.99 | 0.99 |  | MDD | 0.0034 | 0.9175 |  |
|  | OCD | -0.014 | 0.87 | 0.96 |  | OCD | 0.0791 | 0.3104 |  |
|  | PTSD | -0.072 | 0.17 | 0.49 |  | PTSD | -0.038 | 0.4088 |  |
|  | Schizophrenia | 0.011 | 0.76 | 0.94 |  | Schizophrenia | 0.0054 | 0.8642 |  |
|  | Tourette’s | -0.0010 | 0.99 | 0.99 |  | Tourette’s | 0.0649 | 0.3227 |  |
| CT3 | ADHD | -0.0086 | 0.87 | 0.96 |  | | | | |
|  | **Alcohol Dependence** | **-0.350** | **3.00E-04** | **0.012** |  |  |  |  |  |
|  | Anorexia Nervosa | -0.0072 | 0.88 | 0.96 |  |  |  |  |  |
|  | Anxiety | -0.265 | 0.051 | 0.28 |  |  |  |  |  |
|  | Autism | -0.032 | 0.69 | 0.91 |  |  |  |  |  |
|  | Bipolar | 0.020 | 0.66 | 0.91 |  |  |  |  |  |
|  | Cannabis | -0.040 | 0.46 | 0.77 |  |  |  |  |  |
|  | MDD | -0.021 | 0.59 | 0.88 |  |  |  |  |  |
|  | **OCD** | **0.22** | **0.0091** | **0.078** |  |  |  |  |  |
|  | PTSD | -0.037 | 0.45 | 0.77 |  |  |  |  |  |
|  | Schizophrenia | 0.017 | 0.61 | 0.89 |  |  |  |  |  |
|  | Tourette’s | 0.077 | 0.26 | 0.58 |  |  |  |  |  |

Abbreviations: GIBN=genetically informed brain network; CT=cortical thickness derived GIBNS; r_g_=genetic correlation; ADHD=Attention Deficit Hyperactivity Disorder; MDD=Major Depressive Disorder; P_FDR_ False Discovery Rate corrected significance value.
